# Intranasal NS1-truncated live attenuated canine influenza vaccine confers superior protection compared to inactivated vaccine in beagles

**DOI:** 10.1186/s13567-025-01624-7

**Published:** 2025-09-25

**Authors:** Jaehyun Hwang, Sun-Woo Yoon, Eulhae Ga, Jaeseok Choi, Suyun Moon, Eunseo Bae, Hyeongcheol Yun, Dohyeok Yu, Hye Kwon Kim, Jung-Ah Kang, Minjoo Yeom, Jong-Woo Lim, Dae Gwin Jeong, Xing Xie, Daesub Song, Woonsung Na

**Affiliations:** 1 College of Veterinary Medicine, Chonnam National University, Gwangju, 61186 Republic of Korea; 2 Dental Research Institute, School of Dentistry, Seoul National University, Seoul, 03080 Republic of Korea; 3 Department of Biological Sciences and Biotechnology, Andong National University, Andong, 36729 Republic of Korea; 4 Department of Oral Microbiology and Immunology, School of Dentistry, Seoul National University, Seoul, 03080 Republic of Korea; 5 Department of Biological Sciences and Biotechnology, College of National Sciences, Chungbuk National University, Cheongju, 28644 Republic of Korea; 6 Bionanotechnology Research Center, Korea Research Institute of Bioscience and Biotechnology, Daejeon, 34141 Republic of Korea; 7 Department of Virology, College of Veterinary Medicine and Research Institute for Veterinary Science, Seoul National University, Seoul, 08826 Republic of Korea; 8 Key Laboratory for Veterinary Bio-Product Engineering, Ministry of Agriculture and Rural Affairs, Institute of Veterinary Medicine, Jiangsu Academy of Agricultural Sciences, Nanjing, 210014 China; 9 Interdisciplinary Graduate Program in Genetic Engineering, Seoul National University, Seoul, 08826 Republic of Korea

**Keywords:** Canine influenza virus (H3N2), live attenuated influenza vaccine (LAIV), NS1-truncated vaccine, intranasal vaccine, mucosal immunity, beagle dog model, sterile immunity

## Abstract

Canine influenza virus (CIV) H3N2 continues to circulate among companion animals, posing a zoonotic risk due to its potential for cross-species transmission. However, currently available inactivated vaccines offer limited mucosal immunity and suboptimal protection. Here, we developed a novel intranasal live attenuated CIV H3N2 vaccine carrying a truncated nonstructural protein 1 (NS1) gene and evaluated its safety, immunogenicity, and protective efficacy in beagle dogs. The NS1-truncated LAIV was well-tolerated and induced robust mucosal and systemic immune responses, including high titers of virus-specific secretory IgA. Following challenge with virulent CIV H3N2 at 120 days post-vaccination, LAIV-immunized dogs exhibited complete clinical protection and minimal viral shedding, whereas dogs receiving the inactivated vaccine showed moderate disease signs. These findings demonstrate that the NS1-truncated LAIV confers superior protection compared to conventional vaccines and represents a promising next-generation platform for canine influenza control within a One Health framework.

**Supplementary Information:**

The online version contains supplementary material available at 10.1186/s13567-025-01624-7.

## Introduction

Influenza A viruses (IAVs) are highly transmissible pathogens that infect a wide range of avian and mammalian hosts, contributing to substantial disease burden in both humans and animals worldwide [1]. In companion animals, particularly dogs, cross-species transmission of IAV has led to the emergence of canine influenza viruses (CIVs), which cause acute and highly contagious respiratory illness often referred to as “dog flu” [2, 3]. CIV is a key component of the canine infectious respiratory disease complex, and nearly all naïve dogs exposed to the virus develop clinical signs including Nasal discharge, coughing, and fever. Despite the high morbidity observed in outbreak settings, most infections are self-limiting, with a relatively low case-fatality rate estimated at 1–5% [4].

To date, two primary subtypes of CIV have been identified: H3N8 and H3N2. The H3N8 subtype emerged from an equine influenza virus and was first detected in racing greyhounds in the United States in 2004, subsequently spreading throughout the U.S. canine population during the following decade [5]. In contrast, the H3N2 subtype originated from an avian influenza virus and was first isolated from dogs in South Korea in 2007 [6]. This Lineage emerged in East Asia around 2005 and has since become enzootic in canine populations across South Korea and China, later being introduced into the United States in 2015, where it caused multi-state outbreaks [7]. Since then, H3N2 CIV has become the predominant strain circulating among canine populations in East Asia and North America, whereas H3N8 CIV has not been detected since 2018 and is now considered to be no longer circulating. Notably, unlike H3N8, which appears largely restricted to dogs, H3N2 CIV has demonstrated interspecies transmission, including infection of domestic cats with unique clinical outcomes [8–10]. Given the high global population of companion dogs and their close contact with humans, the capacity of H3N2 CIV to breach species barriers raises concern about zoonotic spillover and warrants ongoing surveillance.

The sustained circulation and evolution of H3N2 CIV in dogs have raised legitimate concerns regarding its zoonotic potential [11–14]. Although no human cases of CIV infection have been confirmed to date, multiple virological studies suggest that cross-species transmission is biologically plausible—particularly for the H3N2 subtype. Originally, H3N2 CIV-derived avian influenza virus-exhibited preferential binding to α2,3-linked sialic acid receptors, typical of avian-adapted strains. During more than a decade of adaptation in dogs, H3N2 CIV has acquired several human-like traits, including the additional ability to bind α2,6-linked sialic acid receptors, enhanced Hemagglutinin acid stability, efficient replication in human airway epithelial cells, and airborne transmissibility in ferret models with 100% efficiency [13]. These findings suggest that H3N2 CIV may be approaching the phenotypic threshold for human infection and transmission.

Although there are no confirmed cases of zoonotic influenza viruses originating from dogs and subsequently infecting humans or other species, dogs are susceptible to a broad range of influenza A viruses—including avian, human, and swine strains—and have been found to harbor reassortant viruses such as H3N1 and H3N2 variants carrying gene segments of diverse origin [15–17]. These findings suggest that dogs may serve as potential intermediate hosts with the capacity for influenza virus reassortment, although their role in zoonotic emergence remains under investigation. The anatomical similarity between canine and human respiratory tracts, especially the shared distribution of α2,3 and α2,6 sialic acid receptors, further facilitates interspecies transmission [18]. Given the widespread cohabitation of pet dogs and humans, prior evidence of interspecies influenza jumps, and the immunological naïveté of the human population to canine-origin influenza, public health authorities have emphasized the importance of continuous monitoring of CIV as a potential zoonotic threat [19, 20]. As such, dogs may serve as intermediary hosts in the mammalian adaptation of avian influenza viruses, underscoring the need for robust surveillance and risk assessment strategies.

To mitigate the spread of CIV, inactivated (killed) vaccines have been developed for both the H3N8 and H3N2 subtypes, including bivalent formulations targeting both lineages [21]. These vaccines are commercially available in several countries, including the United States, South Korea, and China. However, routine vaccination is not widely implemented across all regions or dog populations. CIV vaccination is generally recommended for dogs at increased risk of exposure—such as those housed in kennels, shelters, or boarding facilities—as opposed to healthy household pets without high-risk exposures. While these vaccines have shown efficacy in reducing clinical signs, viral replication, and shedding, their overall protective performance remains suboptimal. In particular, current inactivated CIV vaccines predominantly induce systemic humoral responses, but fail to elicit robust mucosal or cellular immunity—critical components for respiratory virus defense [22]. This limitation compromises their ability to prevent infection and transmission at the primary site of viral entry.

Although both H3N8 and H3N2 CIVs share the H3 subtype designation, serological studies have consistently demonstrated minimal cross-protection due to significant antigenic divergence between the two subtypes [23]. Moreover, within, the H3N2 CIV lineage, continuous antigenic drift has been associated with the gradual erosion of vaccine efficacy, even among strain-matched inactivated formulations [24]. These limitations, together with the restricted duration of immunity and inadequate mucosal response induced by conventional vaccines, underscore the urgent need for next-generation vaccines capable of providing broader, more durable protection and improved mucosal immune activation.

Live attenuated influenza vaccines (LAIVs) represent a promising next-generation platform for the control of CIV, offering key immunological advantages over inactivated formulations. Multiple types of LAIVs have been developed, including temperature-sensitive (ts), cold-adapted (ca), and replication-defective viruses. These strategies typically attenuate viral replication based on environmental conditions or intrinsic genetic deletions. In contrast, NS1-truncated LAIVs represent a mechanistically distinct platform. The nonstructural protein 1 (NS1)—a critical antagonist of host type Ⅰ interferon signaling—is genetically truncated to impair viral immune evasion [25–27]. While retaining replication competence, NS1-truncated viruses are highly attenuated in vivo due to increased susceptibility to host innate immune responses [28]. This mechanism enables robust immune stimulation while maintaining a favorable safety profile, making NS1-truncation a powerful strategy for rational vaccine design.

Administered intranasally, LAIVs mimic natural infection and stimulate a broad spectrum of immune responses, particularly mucosal and humoral immunity, and potentially cellular immunity as demonstrated in other models [29–31]. This contrasts with inactivated vaccines, which primarily induce systemic strain-specific antibodies and offer limited mucosal or T-cell-mediated protection. The ability of LAIVs to elicit strong local IgA responses at the site of viral entry—the upper respiratory tract—can significantly limit viral replication and reduce transmission [30, 32, 33]. Furthermore, LAIVs often demonstrate greater cross-protective potential against antigenically drifted strains due to their broader immunogenic profile [29, 34].

Previous studies, including our own, have shown that NS1-truncated influenza viruses induce strong mucosal and cellular immunity and protect effectively in various animal models [28, 35]. Notably, our prior work demonstrated that an H3N2 CIV strain encoding a naturally truncated NS1 protein elicited superior immune responses and protection in mice compared to an inactivated vaccine [35]. These data collectively support the continued development of NS1-truncated LAIVs as a rational and potentially transformative strategy for canine influenza control.

Based on this rationale, we conducted a preclinical evaluation of a novel NS1-truncated LAIV candidate for canine H3N2 influenza. The primary objective was to assess its safety, immunogenicity, and protective efficacy in beagle dogs under controlled experimental conditions, using direct comparison with a currently licensed inactivated CIV vaccine. Key outcome measures included clinical signs, systemic and mucosal immune responses, viral shedding, and pulmonary pathology following virulent challenge.

We hypothesized that the NS1-truncated LAIV would be well-tolerated and elicit more robust immune responses—particularly mucosal and cross-protective immunity—compared to the inactivated vaccine. Consistent with this hypothesis, our findings demonstrated that LAIV-immunized dogs exhibited no clinical signs or viral shedding post-vaccination, and generated significantly stronger humoral and mucosal antibody responses. Upon challenge, LAIV conferred superior protection with reduced clinical illness, minimized viral replication, and negligible lung pathology.

These results support the NS1-truncated LAIV as a safe and highly immunogenic alternative to conventional CIV vaccines, with substantial potential for advancing canine influenza control and contributing to broader One Health preparedness.

## Materials and methods

### Virus strains and vaccine construction

The NS1-truncated live attenuated influenza vaccine (LAIV) was generated using an eight-plasmid reverse genetics system. The internal gene segments (PB2, PB1, PA, NP, M) and the hemagglutinin (HA) and neuraminidase (NA) surface glycoproteins were derived from A/canine/Korea/01/2007 (H3N2) strain (designated as CIV (H3N2)). The non-structural (NS) segment was substituted with that from a naturally occurring equine influenza virus strain, A/equine/Kyonggi/SA1/2011 (H3N8), which encodes a truncated NS1 protein comprising amino acids 1–117.

The eight plasmids were cloned into the bidirectional transcription vector pHW2000 and co-transfected into co-cultured 293 T and MDCK cells using Lipofectamine 3000 (Thermo Fisher). Virus-containing supernatants were harvested 72 h post-transfection and inoculated into the allantoic cavity of 9–11-day-old embryonated chicken eggs to amplify virus stocks. To confirm sequence integrity and truncation of the NS1 gene, the rescued virus underwent full-length Sanger sequencing.

The inactivated CIV control vaccine was prepared using CIV (H3N2) virus propagated in embryonated eggs, inactivated with 0.1% formalin, and the antigen (2^7^ HAU/mL) was mixed with aluminum hydroxide gel (10% v/v final concentration, total volume 500 µL per dog).

### Animals and experimental design

Male beagle dogs were randomly assigned into six experimental groups (*n* = 3 per group): (1) LAIV IN (high),which received 10^3.5^ TCID_50_/dog of LAIV via intranasal inoculation; (2) LAIV IN (low), which received 10^2.5^ TCID_50_/dog of LAIV via intranasal route, (3) Inact-CIV IM, which intramuscularly immunized with inactivated CIV control vaccine (4) CIV IN, which intranasally inoculated with 10^3.5^ TCID_50_/dog of CIV (H3N2), negative control (NC; PBS only, unchallenged), and positive control (PC; unvaccinated but challenged). The CIV IN group was used exclusively for safety assessment.

All animal experiments were approved by the Institutional Animal Care and Use Committee of Chonnam National University (CNU IACUC-YB-2024–51), and conducted under the supervision of licensed veterinarians in ABSL-2 facilities of the College of Veterinary Medicine. Dogs were humanely handled in accordance with ethical guidelines.

Clinical signs were monitored daily from day -1 to 9 post-vaccination (dpv) and from day 0 to 14 post-challenge (dpc), including nasal/ocular discharge and coughing scored as 0 = absent, 1 = moderate, 2 = severe; and fever scored as 0 =  < 40.5 °C or 2 =  ≥ 40.5 °C. Blood samples were collected on dpv 21 and 42. Nasal swabs were collected from dpv 0–8 and dpc 0–11 and additionally at dpv 21.

### In silico antigenic and structural analysis

HA, NA, and NP protein sequences of the LAIV strain (CIV (H3N2) backbone) and recent circulating CIV isolates (collected 2020.04 – 2023.12 from NCBI Virus database, filtered as 2020.04.01–2025.04.01) were aligned to generate consensus sequences. Epitope hotspot positions and ± 5 residue flanks were identified using Jalview and sequence conservation was assessed.

Protein structure predictions for LAIV and consensus CIV antigens were performed using AlphaFold2. B-cell epitope probability and accessibility were analyzed using BepiPred 2.0, including surface exposure (exposed/buried), relative surface accessibility (RSA), secondary structure predictions (helix, sheet, coil), and epitope probability scores.

Predicted CD8 + T cell epitopes in the NP protein were screened by identifying peptides containing P2 anchor residues (L, M, I) and P9 anchor residues (F, L,V), based on binding motif data described in NetCTL and NetMHCpan literature [36–38]. Twelve such candidate epitopes were selected. Structural comparisons and mutation mapping were conducted using PyMOL, and RMSD values were calculated to assess conformational stability.

### Safety and attenuation assessment

Safety of the LAIV was evaluated in vaccinated and virus-inoculated groups by monitoring clinical signs and measuring viral shedding from dpv 0 to 8. Clinical signs were assessed above criteria. Nasal swabs were collected daily and supernatants were serially diluted for TCID assay using MDCK cells in 96-well plates. After 72 h, cytopathic effect was observed and endpoint titers were calculated using the Reed-Muench method [39].

### Humoral and mucosal immune response evaluation

Serum samples collected at dpv 21 and 42 were analyzed for CIV-specific IgG antibodies using ELISA, with plates coated with inactivated CIV antigen. Endpoint titers were determined by the highest dilution with OD greater than mean plus three standard deviations of negative control.

Hemagglutination inhibition (HI) assays used 0.5% chicken red blood cells and 4 HAU of CIV antigen per well. HI titers were recorded as the highest serum dilution that inhibited hemagglutination. Serum neutralization (SN) assays involved mixing serially diluted inactivated serum with 100 TCID_50_ of CIV and applying to MDCK monolayers; the highest dilution preventing cytopathic effect (CPE) was recorded.

Nasal swabs collected at dpv 21 were evaluated for mucosal IgA and IgG by ELISA using similar procedures as for serum, and data were expressed as endpoint titers. For antibody detection, goat polyclonal antibodies (pAb) to dog IgG-HRP and dog IgA-HRP (Abcam) were used as secondary antibodies.

### Protective efficacy and virological assessment post-challenge

Dogs were challenged with 10^6^ TCID_50_ of wild-type CIV via intranasal route at 120 dpv. Nasal swabs were collected daily from dpc 0 to 11 to assess viral replication.

Infectious viral titers were determined using TCID_50_ assay in MDCK cells. For viral RNA quantification, RT-qPCR targeting the matrix (M) gene was performed using Fast Virus 1-Step Master Mix (Thermo Fisher Scientific) with primers: forward (5′-CATGGARTGGCTAAAGACAAGACC-3′), reverse (5′-AGGGCATTTTGGACAAAKCGTCTA-3′), and probe (FAM-5′-ACGCTCACCGTGCCCAGT-3′-BHQ1). Viral genome copies were quantified by a standard curve of in vitro transcribed RNA.

### Histopathological and immunohistochemical evaluation

At dpc 14, one dog per group with median cumulative clinical score was humanely euthanized. The right cranial and middle lung lobes were fixed in 10% neutral-buffered formalin, paraffin embedded, and sectioned for H&E and IHC staining. Histopathological lesions were scored semi-quantitatively for severity (0 = no lesions, 1 = minimal, 2 = mild, 3 = moderate, 4 = severe) and extent (0 = none, 1 =  < 20%, 2 = 20–50%, 3 =  > 50% of a lobe, 4 = diffuse bilateral). Composite lesion scores were calculated by multiplying severity and extent [40].

IHC staining used anti-influenza NP monoclonal antibody with HRP-conjugated secondary antibody and DAB substrate to visualize viral antigen. NP-positive cells were semi-quantitatively scored as: 0 = none, 1 =  < 20 positive cells, 2 = 20–50 cells, 3 = 51–100 cells, 4 =  > 100 cells in representative tissue regions.

### Statistical analysis

All quantitative data are presented as mean ± SEM. Comparisons among groups were performed using Kruskal–Wallis test with Dunn’s post hoc correction for endpoint titer and cumulative scoring data, or two-way repeated-measures ANOVA with Dunnett’s multiple comparison test for dilution and time-course data. Significance was defined as *P* < 0.05 (* *P* < 0.05, ** *P* < 0.01, *** *P* < 0.001, **** *P* < 0.0001). All analyses were conducted using GraphPad Prism 9 (GraphPad Software).

## Results

### In silico antigenic evaluation of NS1-truncated LAIV against recent circulating CIV strains

To generate an attenuated live vaccine candidate, we employed the NS segment derived from A/equine/Kyonggi/SA1/2011 (H3N8) (hereafter referred to as SA1), which contains a 23-nucleotide deletion that induces a frameshift and a premature stop codon, resulting in a 117-amino-acid NS1 protein. In our previous study, this segment was inserted into the A/Puerto Rico/8/1934 (H1N1) backbone and shown to confer significant attenuation in mice by impairing suppression of RIG-Ⅰ-mediated innate immunity [41]. Building on this strategy, we subsequently generated a recombinant A/canine/Korea/01/2007 (H3N2) virus (hereafter referred to as CIV (H3N2)) harboring the same NS segment and confirmed its attenuation and immunogenicity in the murine model [35]. The LAIV candidate used in the present study is this same recombinant virus. Here, we extended its evaluation to the target species—beagle dogs—to assess its safety, immunogenicity, and protective efficacy under controlled experimental conditions.

**Figure 1:**
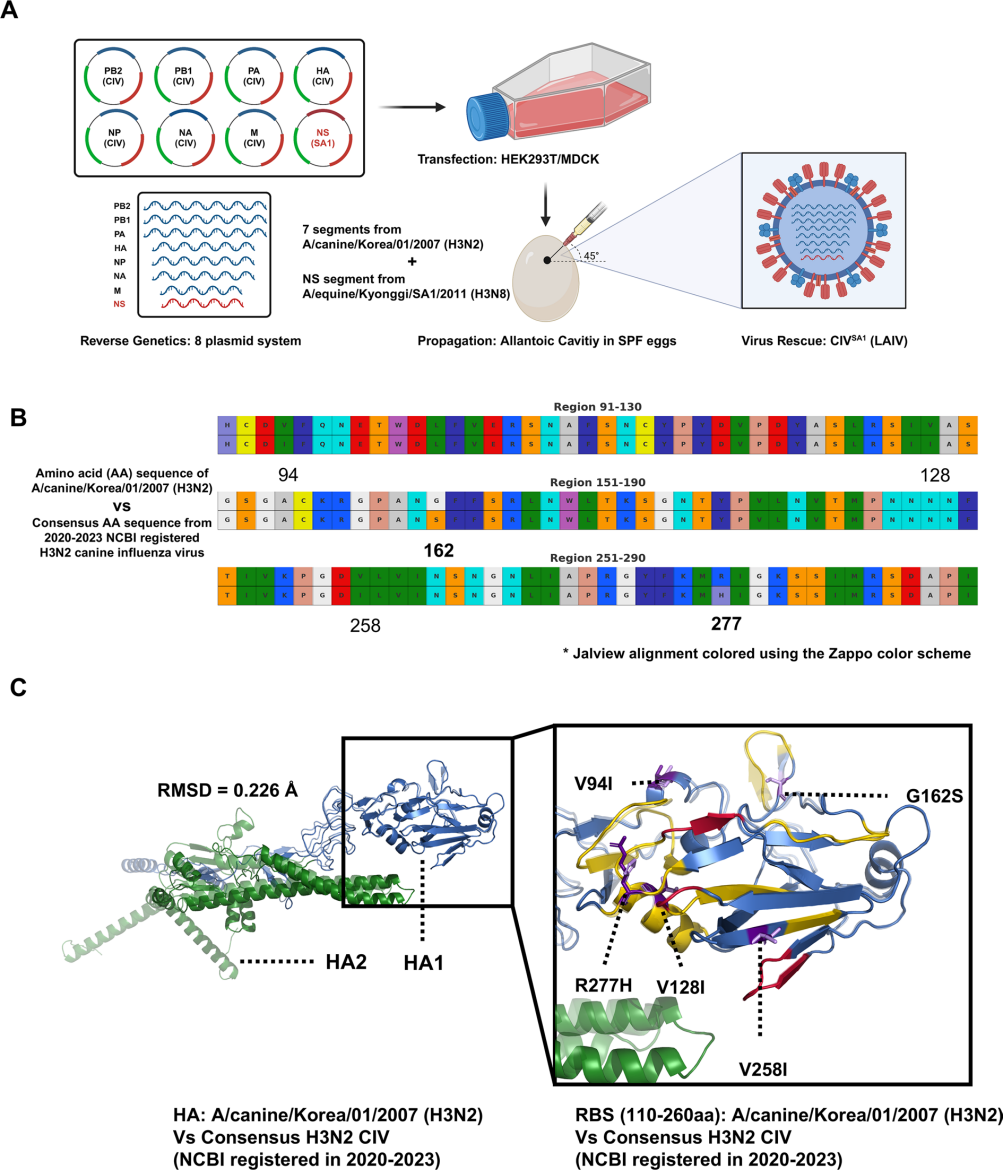
**Genetic and structural characterization of the HA protein in the LAIV compared with circulating H3N2 Canine influenza viruses**.** A** Schematic diagram of LAIV development. The NS1-truncated virus was generated using the 8-plasmid reverse genetics system by replacing the native NS segment of A/canine/Korea/01/2007 (H3N2) with an Naturally 23-nt deleted NS segment derived from A/equine/Kyonggi/SA1/2011 (H3N8). Illustration created with BioRender. **B** Amino acid alignment of HA proteins from A/canine/Korea/01/2007 (H3N2) and the 2020–2023 consensus H3N2 CIV strains (NCBI-registered). Sequence comparison was visualized in Jalview using the Zappo color scheme, which classifies amino acids based on their physicochemical differences. Substitutions in epitope hotspot regions are annotated, with functionally relevant changes shown in bold. **C** Structural comparison of HA proteins modeled using AlphaFold2 and rendered with PyMOL. The consensus H3N2 CIV HA (transparent) is overlaid with the LAIV HA (solid). Domains are color-coded: HA1 (residues 1–328, blue), HA2 (residues 329–566, green), epitope-associated loop and helix structures—130-loop (135–138), 190-helix (188–190), and 220-loop (221–228)—are shown in red. Antigenic sites A–E are shown in gold (site A: 121–137, 140–146; B: 155–160, 188–198; C: 53–55, 275–278; D: 96–105, 171–172; E: 62–64, 78–83, 260–262). Substituted residues in epitope hotspots are represented as sticks (CIV in purple, consensus H3N2 CIV in light purple) with directional annotations

To ensure broad protective potential, we evaluated the antigenic compatibility of the LAIV candidate with currently circulating H3N2 CIV strains. Using the CIV (H3N2) strain as the backbone, the recombinant H3N2 CIV strain with the SA1 NS segment was constructed (Fig. 1A). We performed a multi-layered in silico analysis of epitope conservation and structural alignment across HA, NA, NP, and NS1 proteins. These analyses compared the LAIV strain with: (1) circulating H3N2 CIVs registered in NCBI between 2020–2025; (2) historical H3N8 CIVs (2010–2018); and (3) the parental A/canine/Korea/01/2007 (H3N2) strain.

For the HA protein, five amino acid substitutions were identified within defined epitope hotspots between the LAIV strain and recent CIV consensus HA (Fig. 1B). Epitope hotspot regions were selected based on known functional domains, and mutations within ± 5 flanking residues were listed in Additional file 1. Using BepiPred 2.0, we evaluated the potential impact of these substitutions on epitope accessibility and immunogenicity (Additional file 2). Most of the mutations retained or enhanced structural accessibility, with limited evidence of potential antibody evasion. Notably, no substitutions showed concurrent decreases in surface exposure, relative solvent accessibility (RSA), and epitope probability.

The structural comparison of the LAIV and consensus HA proteins, visualized by PyMOL after AlphaFold2-based modeling, confirmed that the mutated residues did not disrupt the overall topology of the antigenic surface (Fig. 1C). These findings support the hypothesis that the LAIV-derived HA maintains epitope recognition potential for cross-reactivity.

For the Na protein, a similar epitope-centered approach was applied. Five substitution sites within or near predicted B-cell epitope regions were analyzed using BepiPred 2.0 (Additional files 3–4). Although certain mutations such as H155Y and N328D suggested reduced accessibility and epitope potential, other G147S mutation showed increased exposure and structural features favorable for antibody recognition. These structural alterations were further validated by 3D comparison of NA proteins via PyMOL (Additional file 5).

T-cell epitope conservation in the NP protein was assessed based on predicted DLA-88-presented peptide motifs. Despite one substitution, all potential immunodominant regions were structurally conserved (Additional file 6). Additional file 7 visualizes these regions and one mutation site, confirming minimal disruption to predicted T-cell epitopes.

Altogether, this in silico evaluation suggest that the NS1-truncated LAIV strain maintains strong structural and immunological compatibility with circulating H3N2 CIV strains. The majority of substitutions do not alter the accessibility or antigenicity of key B- and T-cell epitope regions, indicating the vaccine’s potential for broad cross-protection. Specifically, among the five HA mutations identified, only R277H was predicted to increase epitope accessibility and potential antibody binding, while the remaining four substitutions were structurally buried or immunologically silent. In NA, one central substitution (R338K) and four peripheral substitutions were analyzed. Despite minor variation in epitope exposure—particularly in H155Y and N328D—none of the substitutions occurred within confirmed epitope cores, and the structural alignment yielded a low RMSD (0.179), indicating overall structural conservation. The NP protein contained a single substitution (R293K), located outside the ± 5 residue range of any predicted DLA-88–binding T-cell epitope. All 12 candidate T-cell epitopes were fully conserved between the vaccine and consensus strains, supporting the preservation of cytotoxic T lymphocyte recognition.

To evaluate the cross-reactive potential beyond H3N2 subtype boundaries, we compared the HA protein of the LAIV strain with the consensus HA sequence of 36 H3N8 CIV strains (2010 and 2018). Despite overall structural conservation, 30 amino acid substitutions were observed in immunodominant regions, particularly within antigenic sites A and B (Additional file 8). AlphaFold2-based structural modeling and PyMOL rendering revealed marked conformational divergence in surface-exposed loops, including the 130-loop and 220-loop, suggesting limited antigenic cross-reactivity between H3N2 and H3N8 CIVs (Additional file 9).

Additionally, sequence alignment of the NS1 protein between SA1 and CIV (H3N2) revealed nine equine strain-specific substitutions, including residues within the linker region between the RNA-binding and effector domains—an area implicated in modulating NS1 structural flexibility and host interaction dynamics (Additional file 10). Notably, a Naturally occurring 23-nucleotide deletion in the SA1 NS segment resulted in a frameshift and the emergence of a distinct C-terminal tail composed of non-canonical residues, potentially contributing to attenuation. Importantly, residues critical for RNA-binding (e.g., R38 and K41) remained conserved.

### Attenuation and clinical safety of the NS1-truncated LAIV in dogs

**Figure 2:**
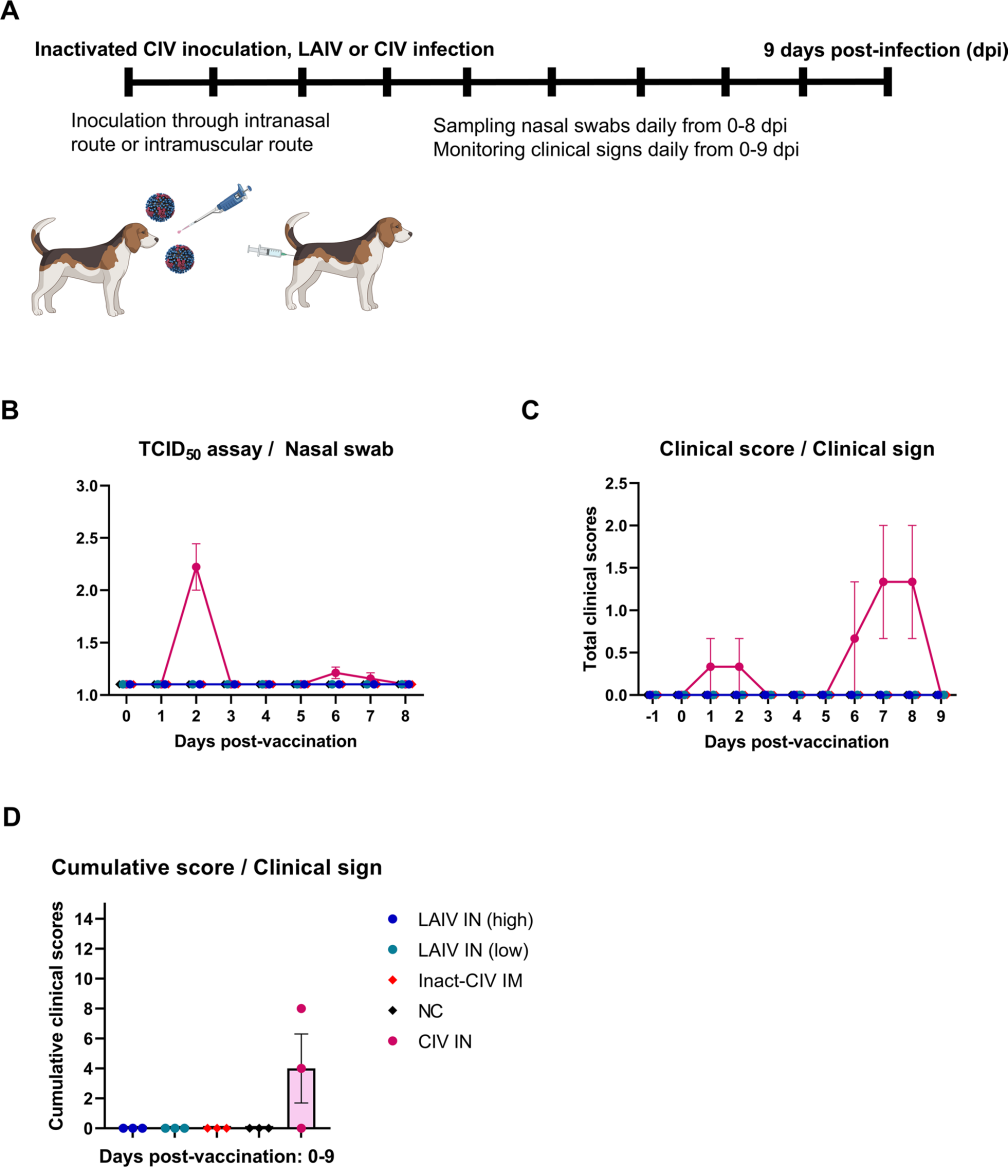
**Clinical safety and attenuation profile of NS1-truncated LAIV following intranasal administration in beagle dogs**. **A** Schematic overview of the experimental design. Beagle dogs (*n* = 3 per group) were assigned to five groups:—LAIV IN (high): 10^3.5^ TCID_50_ of a live attenuated influenza vaccine (LAIV), consisting of 7 segments from A/canine/Korea/01/2007 (H3N2) and NS segment from A/equine/Kyonggi/SA1/2011 (H3N8), administered intranasally.—LAIV IN (low): same LAIV at 10^2.5^ TCID_50_/dog, intranasally.—Inact-CIV IM: 2^7^ HAU/dog of inactivated A/canine/Korea/01/2007 (H3N2) virus formulated with 10% (v/v) aluminum hydroxide gel adjuvant, administered intramuscularly.—CIV IN: 10^3.5^ TCID_50_/dog of wild-type A/canine/Korea/01/2007 (H3N2), administered intranasally.—NC (negative control): PBS only, intranasally. All vaccinated groups were challenged with wild-type CIV (H3N2) at 120 days post-vaccination. **B** Viral shedding was assessed by collecting Nasal swabs from 0 to 8 days post-infection (dpi) and determining infectious titers using TCID₅₀ assay. **C** Daily clinical scores were recorded from dpi –1 to 9 based on nasal and ocular discharge, cough, and fever (≥ 40.5 °C), following a standardized scoring system (see Additional file 11). **D** Cumulative clinical scores calculated for each individual dog across the entire observation period. No infectious virus was detected from nasal swabs and no clinical signs were observed in any of the vaccinated groups or PBS inoculated group (LAIV high-dose, LAIV low-dose, inact-CIV, and NC). These findings indicate that viral shedding and clinical symptoms were restricted to the wild-type CIV group. Each symbol represents an individual dog. Horizontal bars represent group means; error bars indicate standard error of the mean (SEM). Statistical comparisons were performed using (**B, C**) nonparametric two-way ANOVA with Dunnett’s multiple comparisons test, and (**D**) Kruskal–Wallis test followed by Dunn’s test. *p < 0.05; **p < 0.01; ***p < 0.001; ****p < 0.0001

The attenuation and safety profile of the NS1-truncated LAIV strain was assessed in beagle dogs following intranasal vaccination. Fig. 2A outlines the experimental design. Dogs (*n* = 3 per group) were assigned to one of the following groups: LAIV high-dose (10^3.5^ TCID_50_ in 500 µL, IN), LAIV low-dose (10^2.5^ TCID_50_ in 500 µL, IN), inactivated CIV mixed with aluminum hydroxide gel adjuvant (2^7^ HAU in 500 µL, IM), wild-type CIV infection control (10^3.5^ TCID_50_ in 500 µL, IN without prior vaccination), and PBS as a negative control. Vaccinated groups (LAIV and inactivated CIV) were challenged 120 days post-vaccination with wild-type A/canine/Korea/01/2007 (CIV H3N2), whereas the wild-type CIV group was directly infected without prior immunization.

Clinical signs were monitored daily from 1 day before infection to 9 days post-infection (dpi), including nasal/ocular discharge (0–2), coughing (0–2), and fever (0 or 2 if ≥ 40.5 °C), as defined in Additional file 11. To assess vaccine transmissibility, Nasal swabs were collected daily from 0 to 8 dpi and subjected to TCID_50_ assay (Fig. 2B).

No detectable virus was recovered from the nasal swabs of LAIV-vaccinated dogs, confirming attenuation and non-transmissibility of the vaccine virus. In contrast, viral shedding was consistently detected in the wild-type CIV group. Daily clinical scores (Fig. 2C) showed complete absence of symptoms in both LAIV groups. In contrast, mild signs were observed in the inactivated CIV group, while the wild-type CIV group exhibited more severe clinical illness.

Cumulative clinical scores (Fig. 2D) further demonstrated these differences: 2 out of 3 wild-type-infected dogs developed noticeable symptoms, while all LAIV-vaccinated animals remained asymptomatic throughout the study.

These results indicate that the NS1-truncated LAIV is clinically safe, non-replicative in the upper respiratory tract, and significantly attenuated compared to wild-type CIV, making it a promising intranasal vaccine candidate for canine use.

### NS1-truncated LAIV induces dose-dependent systemic humoral immunity in beagle dogs

**Figure 3:**
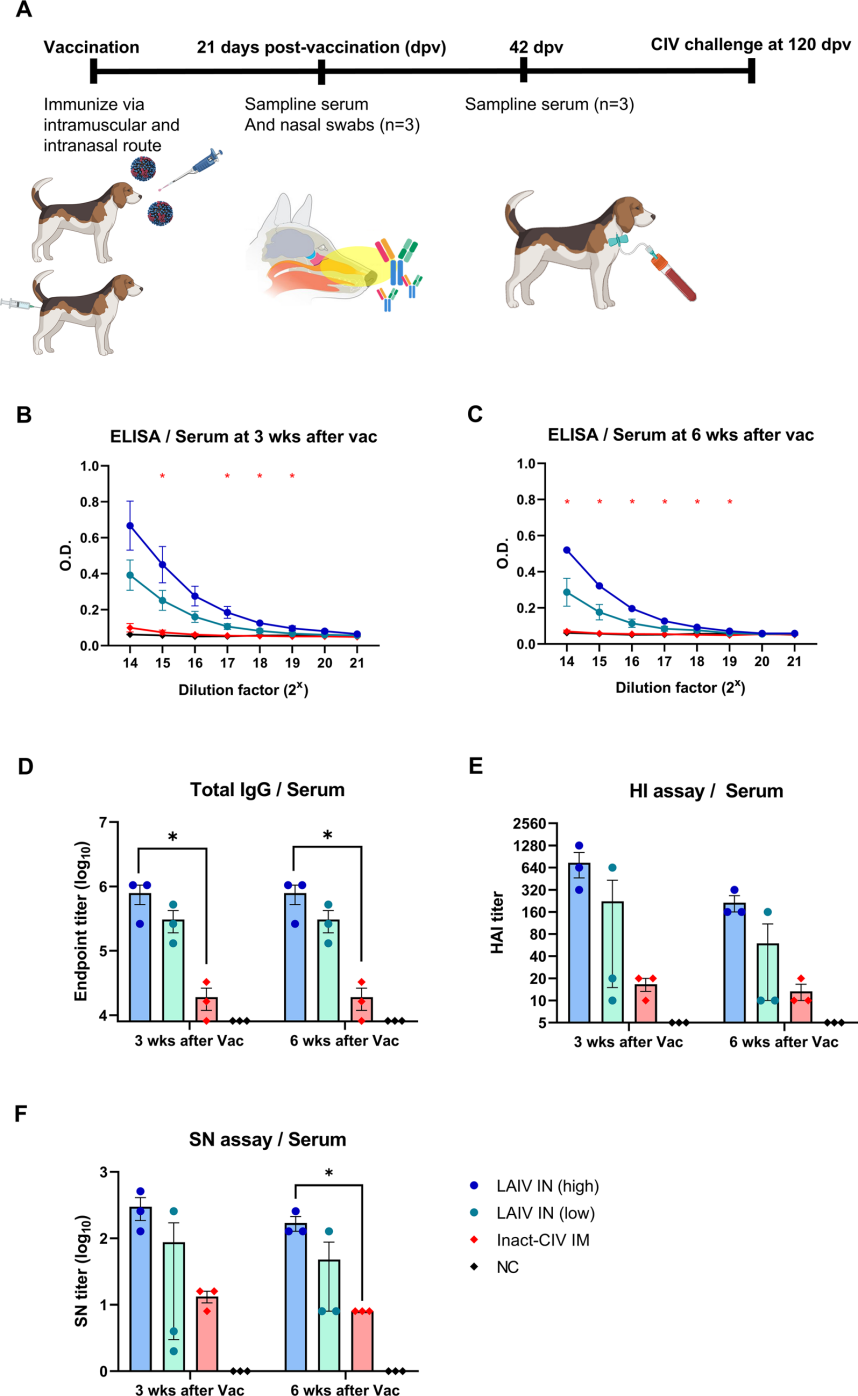
**Systemic humoral immune responses induced by intranasal NS1-truncated LAIV in beagle dogs**. **A** Schematic timeline of vaccine administration and sample collection. Beagle dogs (*n* = 3 per group) were assigned to four groups:—LAIV IN (high): 10^3.5^ TCID_50_ of a live attenuated influenza vaccine (LAIV), consisting of 7 segments from A/canine/Korea/01/2007 (H3N2) and NS segment from A/equine/Kyonggi/SA1/2011 (H3N8), administered intranasally.—LAIV IN (low): same LAIV at 10^2.5^ TCID_50_/dog, intranasally.—Inact-CIV IM: 2^7^ HAU/dog of inactivated A/canine/Korea/01/2007 (H3N2) virus formulated with 10% (v/v) aluminum hydroxide gel adjuvant, administered intramuscularly.—NC (negative control): PBS only, intranasally. Blood samples were collected at 21 and 42 days post-vaccination. **B, C** Anti-CIV IgG levels were measured in serum samples collected on days (**B**) 21 and (**C**) 42 by ELISA. **D** Endpoint IgG titers were calculated for individual animals based on the ELISA data at both time points. **E**, **F** Functional antibody responses were assessed at 21 and 42 days post-vaccination using (**E**) hemagglutination inhibition (HI) assay and (**F**) serum neutralization (SN) assay. Each symbol represents one dog. Horizontal lines and error bars denote group means and standard error of the mean (SEM), respectively. Statistical analysis was performed using (**B, C**) nonparametric two-way ANOVA with Dunnett’s multiple comparisons test, and (**D**, **E**, **F**) Kruskal–Wallis test with Dunn’s post hoc test. *p < 0.05; **p < 0.01; ***p < 0.001; ****p < 0.0001

Systemic antibody responses were evaluated by measuring anti-CIV IgG levels in sera collected at 21 and 42 days post-vaccination (Fig. 3A). ELISA results at both time points (Fig. 3B and 3C) showed a clear dose-dependent increase in IgG titers in LAIV-vaccinated groups. Notably, dogs receiving high-dose LAIV via the intranasal route (LAIV IN (high)) exhibited significantly higher anti-CIV IgG titers compared to the inactivated CIV group at both time points.

Endpoint titers calculated for individual animals (Fig. 3D) confirmed this observation, with LAIV IN high consistently inducing significantly higher IgG titers than inactivated CIV on both days 21 and 42 post-vaccination.

Functional antibody activity was assessed using hemagglutination inhibition (HI) assays (Fig. 3E) and serum neutralization (SN) assays (Fig. 3F). While differences in HI titers did not reach statistical significance, a consistent trend was observed, with titers ranked in the order of LAIV IN (high) > LAIV IN (low) > inactivated CIV. A similar pattern was observed in the SN assay at day 21. By day 42, SN titers in the LAIV IN (high) group were significantly higher than those in the inactivated CIV group, indicating enhanced generation of neutralizing antibodies following live attenuated vaccination.

These data demonstrate that the NS1-truncated LAIV elicits a strong, dose-dependent systemic IgG response and promotes superior functional antibody activity compared to the inactivated vaccine platform, particularly in terms of neutralization potency.

### Intranasal LAIV vaccination induces dose-dependent mucosal antibody responses

**Figure 4:**
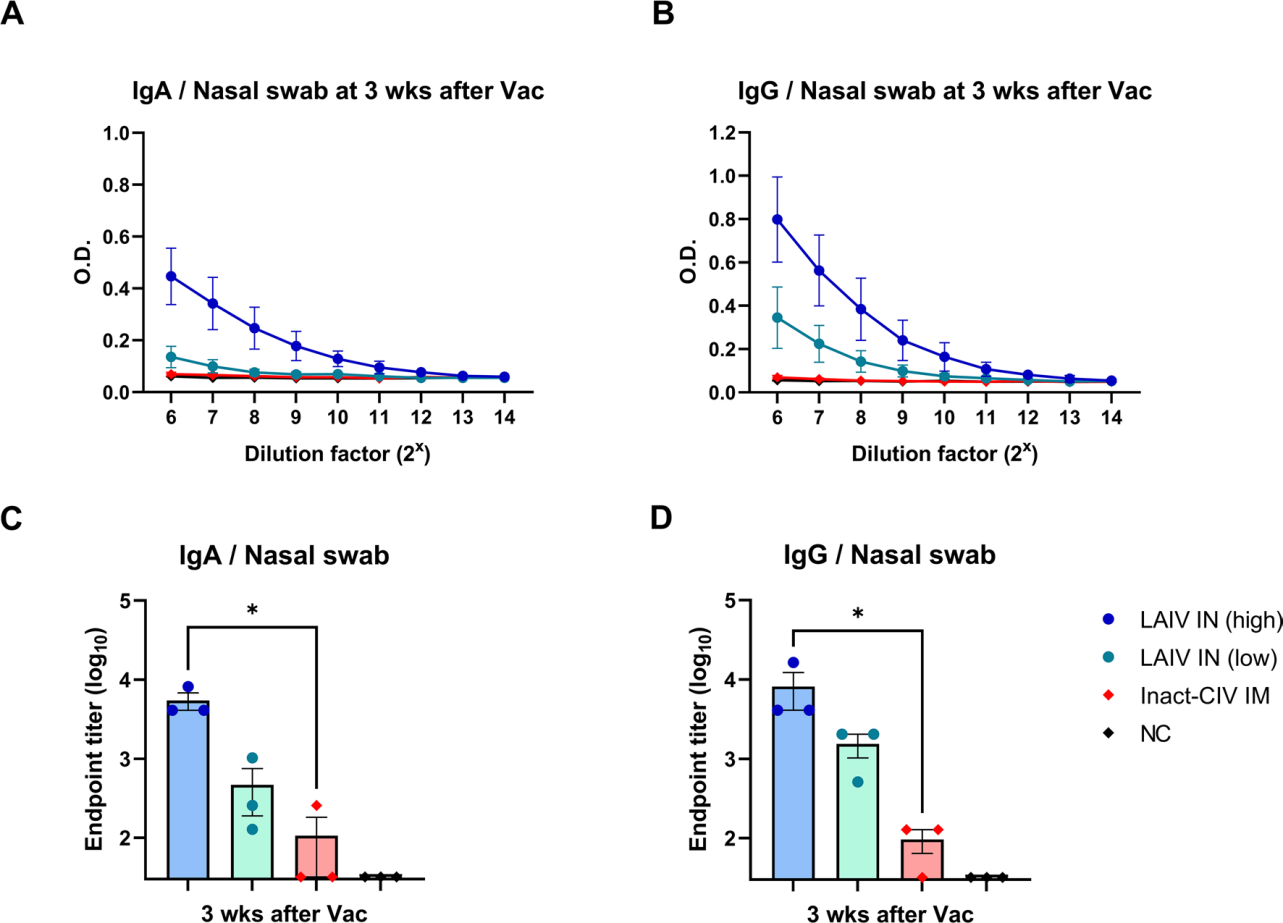
**Mucosal immune responses induced by intranasal NS1-truncated LAIV in beagle dogs.** Nasal swab samples were collected at 21 days post-vaccination to evaluate mucosal antibody responses in beagle dogs (*n* = 3 per group). Beagle dogs were assigned to four groups:—LAIV IN (high): 10^3.5^ TCID_50_ of a live attenuated influenza vaccine (LAIV), consisting of 7 segments from A/canine/Korea/01/2007 (H3N2) and NS segment from A/equine/Kyonggi/SA1/2011 (H3N8), administered intranasally.—LAIV IN (low): same LAIV at 10^2.5^ TCID_50_/dog, intranasally.—Inact-CIV IM: 2^7^ HAU/dog of inactivated A/canine/Korea/01/2007 (H3N2) virus formulated with 10% (v/v) aluminum hydroxide gel adjuvant, administered intramuscularly.—NC (negative control): PBS only, intranasally. **A, B** Anti-CIV IgA (**A**) and Anti-CIV IgG (**B**) levels in nasal swabs were measured by ELISA. (**C**, **D**) Endpoint titers for nasal IgA (**C**) and IgG (**D**) were calculated from individual animals to compare mucosal antibody responses across groups. Each symbol represents an individual dog. Horizontal lines and error bars indicate group means and standard error of the mean (SEM), respectively. Statistical analyses were performed using (**A**, **B**) nonparametric two-way ANOVA with Dunnett’s multiple comparisons test, and (**C, D**) Kruskal–Wallis test with Dunn’s post hoc test. * p < 0.05; ** p < 0.01; *** p < 0.001; **** p < 0.0001

To evaluate mucosal immunity induced by the NS1-truncated LAIV, Nasal swab samples were collected at 21 days post-vaccination and analyzed by ELISA for secretory IgA and IgG levels (Fig. 4A and B). A dose-dependent trend was observed in both antibody isotypes, with LAIV IN (high) inducing the highest levels, followed by LAIV IN (low), and then inactivated CIV. Although optical density values did not differ significantly among groups, mucosal IgA and IgG levels in the inactivated CIV group were comparable to those of the negative control. No statistically significant increase in mucosal IgA or IgG levels was observed in the inactivated CIV group compared to the negative control, suggesting that intramuscular vaccination fails to elicit upper respiratory mucosal immunity.

Endpoint titers calculated for individual animals (Fig. 4C and D) confirmed the dose-dependent pattern observed in raw values. LAIV IN (high) induced significantly higher IgA and IgG titers in nasal swabs compared to the inactivated CIV group, indicating that live attenuated intranasal vaccination is more effective at inducing mucosal antibody responses in the respiratory tract.

### Clinical protection and viral suppression following CIV challenge

**Figure 5:**
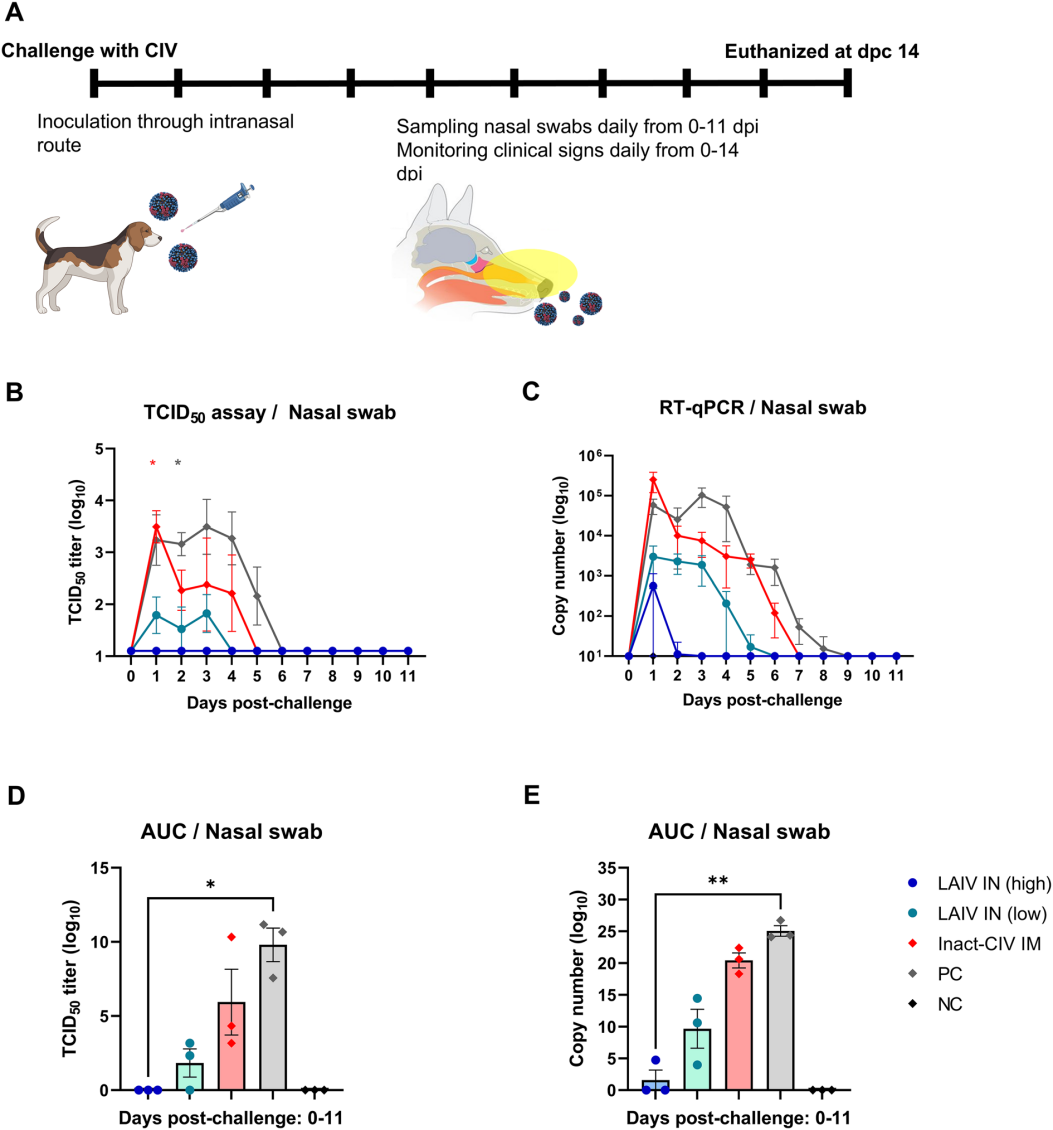
**Protective efficacy of NS1-truncated LAIV against CIV challenge: viral shedding and clinical outcome.** **A** Schematic overview of the challenge protocol, clinical observation period, nasal swab collection schedule, and endpoint necropsy. Beagle dogs (*n* = 3 per group) were previously vaccinated with:—LAIV IN (high): 10^3.5^ TCID_50_ of a live attenuated influenza vaccine (LAIV), consisting of 7 segments from A/canine/Korea/01/2007 (H3N2) and NS segment from A/equine/Kyonggi/SA1/2011 (H3N8), administered intranasally.—LAIV IN (low): same LAIV at 10^2.5^ TCID_50_/dog, intranasally.—Inact-CIV IM: 2^7^ HAU/dog of inactivated A/canine/Korea/01/2007 (H3N2) virus formulated with 10% (v/v) aluminum hydroxide gel adjuvant, administered intramuscularly. At 120 days post-vaccination, dogs were challenged intranasally with wild type A/canine/Korea/01/2007 (H3N2) virus at 10^6 ^TCID_50_/dog.—PC (positive control): unvaccinated but challenge with the same wild-type CIV- NC (negative control): unvaccinated and unchallenged. One dog per group—selected based on median cumulative clinical score—was humanely euthanized on 14 days post-challenge (dpc) for histopathological evaluation. **B** Infectious viral titers in Nasal swab samples collected from dpc 0 to 11 were quantified by TCID₅₀ assay. **C** Viral RNA levels were determined by RT-qPCR targeting the influenza M segment using the same nasal swab samples. **D**, **E** Cumulative viral burden was assessed by calculating the area under the curve (AUC) for individual animals based on (**D**) infectious virus titers and (**E**) viral RNA copy numbers. Each symbol in the graphs represents an individual dog, while horizontal bars and error bars indicate the group mean and standard error of the mean (SEM), respectively. Statistical comparisons were performed using two-way ANOVA with Dunnett’s multiple comparisons test for **(B, C**), and Kruskal–Wallis test with Dunn’s post hoc test for (**D**, **E**). Colored asterisks in (**B**) indicate statistically significant differences between the LAIV IN (high) group and the group represented by the corresponding color. * p < 0.05; ** p < 0.01; *** p < 0.001; **** p < 0.0001

To evaluate the long-term protective efficacy of the NS1-truncated LAIV, vaccinated beagle dogs were challenged intranasally with wild-type CIV (10^6^ TCID_50_/dog) at 120 days post-vaccination. Daily clinical signs were recorded for 14 days post-challenge (dpc), and Nasal swabs were collected from 0 to 11 dpc for quantification of viral load (Fig. 5A).

Clinical scores were assigned based on nasal/ocular discharge, coughing, and fever, as detailed in Additional file 12. LAIV IN (high) dogs displayed no clinical signs throughout the monitoring period. In the LAIV IN (low) group, one dog exhibited transient fever on dpc 1 and 3. In contrast, mild-to-moderate signs were observed in two dogs in the inactivated CIV group, including early fever followed by Nasal discharge and coughing from dpc 6 onward. The positive control (PC) group showed variable clinical signs in all animals.

Viral shedding was assessed using TCID_50_ assays (Fig. 5B) and M gene RT-qPCR (Fig. 5C). LAIV IN (high) dogs showed no detectable infectious virus throughout the sampling period. In the LAIV IN (low), inactivated CIV, and PC groups, viral clearance was observed from dpc 4, 5, and 6, respectively. Area under the curve (AUC) analyses of both infectious viral titers (Fig. 5D) and viral RNA levels (Fig. 5E) demonstrated significantly lower cumulative viral burden in the LAIV IN (high) group compared to PC, with an overall trend of LAIV IN (high) < LAIV IN (low) < inactivated CIV < PC.

These results indicate that NS1-truncated LAIV confers robust clinical protection and significantly limits viral replication in the upper respiratory tract following homologous CIV challenge.

### Attenuation of pulmonary pathology in LAIV-vaccinated animals

**Figure 6:**
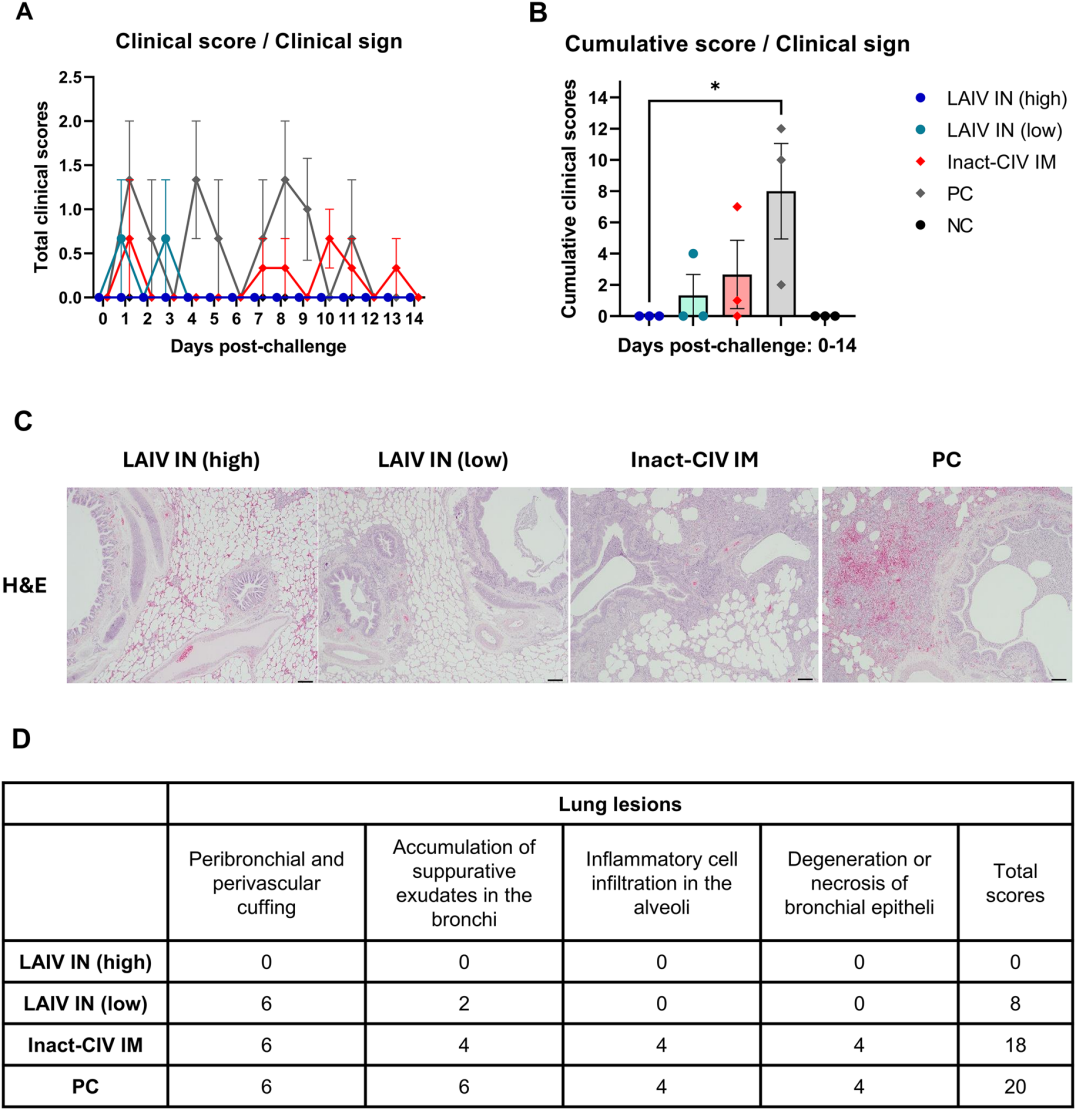
**Clinical and histopathological evaluation of protective efficacy following CIV challenge.** **A**, **B** Beagle dogs (*n* = 3 per group) were previously vaccinated with:—LAIV IN (high): 10^3.5^ TCID_50_ of a live attenuated influenza vaccine (LAIV), consisting of 7 segments from A/canine/Korea/01/2007 (H3N2) and NS segment from A/equine/Kyonggi/SA1/2011 (H3N8), administered intranasally.- LAIV IN (low): same LAIV at 10^2.5^ TCID_50_/dog, intranasally.—Inact-CIV IM: 2^7^ HAU/dog of inactivated A/canine/Korea/01/2007 (H3N2) virus formulated with 10% (v/v) aluminum hydroxide gel adjuvant, administered intramuscularly. At 120 days post-vaccination, dogs were challenged intranasally with wild type A/canine/Korea/01/2007 (H3N2) virus at 10^6^TCID_50_/dog.—PC (positive control): unvaccinated but challenge with the same wild-type CIV.—NC (negative control): unvaccinated and unchallenged. Clinical signs were monitored daily from 0 to 14 days post-challenge (dpc), including nasal/ocular discharge (0–2), coughing (0–2), and fever (0 or 2 if ≥ 40.5 °C). Daily clinical scores are presented in (**A**), and cumulative clinical scores per animal over the observation period are summarized in (**B**). (**C**, **D**) On dpc 14, one representative dog per group (selected based on the median cumulative clinical score) was humanely euthanized. The right cranial and middle lung lobes were collected, fixed in 10% neutral buffered formalin for 48 h, and stained with hematoxylin and eosin (H&E). Lung lesions were evaluated semi-quantitatively based on a dual-criteria system: severity (grade, 0–4) and extent (stage, 0–4). Final histopathology scores were calculated as the product of grade and stage to reflect overall lesion burden (**D**). Each symbol in the graphs represents an individual dog; horizontal lines and error bars denote group means and standard error of the mean (SEM), respectively. Statistical analysis was performed using (**A**) nonparametric two-way ANOVA with Dunnett’s multiple comparisons test, and (**B**) Kruskal–Wallis test with Dunn’s post hoc test * p < 0.05; ** p < 0.01; *** p < 0.001; **** p < 0.0001

To evaluate lung pathology following CIV challenge, daily clinical scores were visualized (Fig. 6A) and summarized as cumulative scores per animal (Fig. 6B). Representative animals from each group—selected based on median clinical score—were euthanized at dpc 14 for histopathological analysis. Lung tissues (right cranial and middle lobes) were processed for H&E staining (Fig. 6C) and immunohistochemistry (IHC) using anti-NP antibody (Additional file 13).

Histopathological scoring based on lesion severity (grade) and extent (stage) was used to calculate total lung lesion scores (Fig. 6D). LAIV IN (high) animals showed minimal tissue damage, followed by LAIV IN (low) and inactivated CIV groups. The PC group exhibited the highest lesion scores, including peribronchiolar inflammation and interstitial cellular infiltration.

IHC analysis revealed no NP-positive cells in lung tissues from any group at dpc 14, suggesting that active viral replication had resolved by the time of sampling.

Together, these findings support the conclusion that NS1-truncated LAIV not only prevents clinical disease and viral shedding but also effectively limits histopathological damage in the lungs, confirming its protective efficacy in a canine influenza challenge model.

## Discussion

Our findings demonstrate that an intranasally administered NS1-truncated live attenuated influenza vaccine (LAIV) confers significantly enhanced immunogenicity and protective efficacy against canine influenza virus (CIV) compared to the conventional inactivated vaccine, while maintaining an excellent safety profile. Using a well-characterized beagle model, we systematically validated that this LAIV platform overcomes several key limitations of current CIV vaccines—most notably their inability to induce mucosal immunity and their limited protective breadth following viral challenge.

The in silico epitope conservation analysis further substantiates the cross-protective potential of the NS1-truncated LAIV platform against recently circulating CIV strains. Structural and immunological comparisons between the LAIV backbone strain (A/canine/Korea/01/2007) and consensus sequences from H3N2 CIV isolates collected between 2020 and 2023 revealed that most amino acid substitutions in the HA and NA proteins were either structurally buried or predicted to be immunologically neutral. Notably, one HA substitution, R277H, was positioned within a central B-cell epitope hotspot and was associated with increased surface accessibility and epitope probability, potentially enhancing antibody recognition [42, 43]. In the NA protein, two substitutions—H155Y and N328D—supported modest reductions in B-cell epitope scores but were located in epitope-flanking regions rather than core sites. Despite these local changes, the NA protein retained a highly conserved global architecture, as indicated by a low RMSD value (0.179) between the LAIV and consensus NA models [44].

With respect to cellular immunity, twelve predicted DLA-88–restricted CD8⁺ T cell epitope candidates in the NP protein were fully conserved between the LAIV and contemporary strains [45]. The only observed mutation, R293K, was located outside of defined epitope regions and their ± 5 residue flanks. This conservation implies that CD8⁺ T cell responses elicited by the LAIV are likely to remain effective against antigenically drifted field strains. Collectively, these in silico findings highlight that the LAIV strain maintains critical epitope integrity across both B- and T-cell targets, supporting its potential as a broadly protective and evolution-resilient vaccine. Such immunological breadth is essential for controlling CIV outbreaks in diverse canine populations and aligns with the One Health imperative of mitigating cross-species transmission risks.

To further delineate the breadth and limitations of inter-subtype cross-reactivity, we extended our in silico evaluation to include H3N8 CIV strains. Structural alignment between the LAIV-derived HA and the H3N8 consensus sequence revealed numerous substitutions in key epitope-bearing regions, particularly in the 130- and 220-loops. These alterations introduced significant conformational changes, as visualized by structural modeling, and are consistent with prior serological data indicating minimal humoral cross-protection between H3N2 and H3N8 subtypes [23].

In addition to antigenic considerations, we examined the safety-related implications of incorporating a 23-nt deleted NS segment derived from the equine-adapted SA1 strain. The SA1 NS segment harbors multiple equine-specific substitutions, some of which localize to functionally relevant regions such as the interdomain linker [46]. Importantly, the 23-nt deletion that underlies the NS1 truncation induces a frameshift, resulting in a premature stop codon at residue 117 and the generation of a novel C-terminal tail absent from wild type NS1. This aberrant sequence lacks critical host interaction motifs—such as the CPSF30-binding region—rendering it functionally deficient [47]. Even if a point mutation were to restore a sense codon at the stop site, the downstream coding frame would remain disrupted, making functional reversion highly unlikely.

Crucially, this truncation is of natural origin, not laboratory-engineered mutation. The SA1-derived NS1 protein was identified in field-isolated equine influenza virus, indicating that this variant was replication-competent in its natural host and has withstood negative selection pressure at the time of isolation. Its presence in nature suggests evolutionary stability, further supporting its biosafety profile as a vaccine backbone. Together, the structural constraint imposed by the frameshift and the field-validated genetic stability significantly reduce the risk of virulence reversion.

The safety profile of our NS1-truncated LAIV represents a meaningful advancement in the field of canine respiratory vaccines. Following intranasal administration, the vaccine strain produced no detectable viral shedding or clinical manifestations in dogs, including those receiving a high-dose formulation. This attenuation is consistent with the well-characterized effects of NS1 truncation, which limits viral replication in vivo by abrogating the virus's ability to antagonize host innate immunity, particularly type Ⅰ interferon responses [25, 26]. The NS1 protein normally functions as a potent interferon antagonist, and its truncation markedly reduces virulence while preserving antigen expression and immunogenicity [27]. Our findings are in line with previous studies demonstrating that influenza viruses engineered with truncated NS1 proteins exhibit highly attenuated phenotypes but remain capable of eliciting protective immune responses [28, 35, 48, 49]. Notably, the complete absence of clinical signs and viral dissemination in LAIV-vaccinated dogs—even at doses tenfold higher than the low-dose group—underscores the safety of this platform, which was comparable to that of inactivated vaccine while retaining the enhanced immunological advantages of live vaccination.

The immunogenic superiority of our NS1-truncated LAIV was clearly demonstrated across multiple measures of systemic antibody responses. Compared to the inactivated vaccine, intranasal LAIV administration elicited significantly higher titers of CIV-specific IgG, hemagglutination inhibition (HI), and serum neutralization (SN) antibodies. These responses were evident as early as three weeks post-vaccination and were sustained through six weeks. Notably, LAIV-vaccinated dogs in the high-dose group exhibited up to 46-fold greater HI titers and approximately 20-fold higher SN titers compared to the inactivated vaccine group. These robust humoral responses are likely to contribute substantially to the enhanced protective efficacy observed following viral challenge.

The superior immunogenicity of the LAIV can be attributed to several factors inherent to live vaccine platforms. First, intranasal administration allows the vaccine virus to replicate locally in the upper respiratory mucosa, mimicking natural infection and promoting the development of both local and systemic immune responses. Second, the attenuated virus retains the capacity to engage innate immune sensors, such as pattern recognition receptors (PRRs), which are critical for the induction of adaptive immunity. Third, the native conformation of viral antigens presented during LAIV replication may better preserve conformational epitopes, thereby improving B-cell receptor engagement and antibody affinity maturation [50].

Importantly, a clear dose-dependent pattern was observed in LAIV-vaccinated animals, wherein higher inoculum levels correlated with stronger antibody responses. This dose-responsiveness offers a practical advantage in vaccine formulation, allowing for potency adjustment without compromising safety. Collectively, these findings underscore the ability of the NS1-truncated LAIV to elicit potent, durable, and functional antibody responses that surpass those of conventional inactivated CIV vaccines.

Perhaps the most striking advantage of the NS1-truncated LAIV platform was its ability to elicit robust mucosal immune responses in the upper respiratory tract—responses that were virtually absent following inactivated vaccine administration [51]. LAIV-vaccinated dogs developed significantly higher levels of CIV-specific secretory IgA and IgG in Nasal swab samples compared to the inactivated vaccine group, with the magnitude of mucosal IgA responses exceeding those of parenterally immunized animals by approximately 50-fold. Notably, mucosal antibody titers in the inactivated vaccine group did not differ significantly from those of unvaccinated controls, underscoring the limitations of intramuscular delivery in generating protective immunity at mucosal surfaces.

The ability of intranasally administered LAIV to induce such strong local antibody responses aligns with established principles in mucosal immunology [30]. Secretory IgA acts as a frontline defense at the site of viral entry, preventing infection through multiple mechanisms: direct neutralization of virions, inhibition of intracellular viral assembly, and immune exclusion via entrapment in mucus [52, 53]. These functions collectively contribute to limiting initial viral colonization and reducing onward transmission [54].

The presence of high titers of mucosal antibodies in LAIV-immunized animals likely played a central role in the rapid viral clearance observed post-challenge, as demonstrated by the significantly lower infectious and genomic viral loads in nasal swabs [30, 55]. These findings reinforce the critical importance of mucosal immunity in controlling respiratory pathogens and position the NS1-truncated LAIV as a superior platform for respiratory virus prevention in companion animals [56].

The protective efficacy of the NS1-truncated LAIV was evident across clinical, virological, and pathological domains following virulent CIV challenge. Dogs immunized with the high-dose LAIV formulation displayed complete protection, exhibiting no clinical signs such as nasal discharge, coughing, or fever throughout the post-challenge period. In contrast, unvaccinated controls consistently developed progressive respiratory symptoms. Partial protection was observed in the low-dose LAIV and inactivated vaccine groups, where some animals showed transient or delayed onset of symptoms. These clinical outcomes followed a dose-dependent pattern consistent with the immunization regimen.

Virological analyses reinforced these findings. Dogs in the high-dose LAIV group maintained significantly lower levels of both infectious viral titers and viral RNA in nasal secretions compared to unvaccinated controls. Although statistical significance was not achieved for comparisons with intermediate groups, a clear stepwise reduction in viral burden was observed, suggesting a correlation between vaccine dose and viral control. In particular, the absence of detectable infectious virus throughout the observation period in high-dose recipients indicates the potential for strong local viral containment [57].

Lung tissue evaluation further supported the immunological trends observed. Representative animals selected from each group for pathological analysis demonstrated minimal pulmonary inflammation in LAIV-vaccinated dogs, while control animals exhibited more pronounced lesions consistent with active viral pneumonia. Although statistical inference was limited by sample size, these findings align with the observed clinical and virological trends [57]. No viral antigen was detected in any of the lung samples by immunohistochemistry at the terminal timepoint, suggesting that viral clearance in the lower respiratory tract was complete by two weeks post-challenge, irrespective of vaccination status.

Taken together, these findings demonstrate that the NS1-truncated LAIV effectively mitigates clinical illness and reduces viral replication in a dose-responsive manner. While certain histological endpoints were limited by experimental design, the consistent reductions in clinical severity and upper respiratory viral burden underscore the vaccine’s protective capacity. This degree of protection is particularly relevant for controlling CIV outbreaks in high-density canine settings, such as shelters or boarding facilities, where rapid transmission is a persistent concern. By curtailing viral shedding and limiting disease progression, this vaccine platform also aligns with broader One Health goals aimed at minimizing the risk of influenza virus adaptation and interspecies transmission [56, 58, 59].

The protective efficacy of the NS1-truncated LAIV observed in this study is likely mediated by the combined effects of humoral and mucosal immune responses. Systemic IgG antibodies provide essential protection by neutralizing virus that disseminates beyond mucosal barriers, while locally secreted IgA in the upper respiratory tract plays a pivotal role in intercepting virus at the site of entry. The high levels of virus-specific mucosal IgA detected following intranasal LAIV administration suggest that this immune component may be critical for the rapid viral clearance and absence of detectable viral shedding observed after challenge. These immune responses—measurable and robust—are consistent with the near-sterilizing immunity achieved in the high-dose LAIV group [60].

From a veterinary and public health standpoint, these findings support a shift in CIV vaccine strategies. Inactivated vaccines, though widely used, require multiple doses and primarily stimulate systemic immunity, which may be insufficient for halting transmission. In contrast, the LAIV described here achieved durable humoral and mucosal immunity with a single intranasal dose, simplifying administration and enhancing outbreak responsiveness—an especially relevant advantage in high-density canine settings such as shelters and boarding facilities.

As canine influenza viruses continue to evolve, the ability of a vaccine to provide broad and durable protection becomes increasingly important. While this study focused on homologous protection, the in silico antigenic analysis supported preservation of key B-cell epitope structures in recent circulating strains. These findings, coupled with the strong functional antibody responses observed in vaccinated animals, suggest that this LAIV platform may be better positioned than inactivated vaccines to accommodate antigenic drift.

Several limitations merit consideration. Although the sample size was sufficient to detect major difference across groups, it may have limited resolution of more subtle immune variations. Protection was evaluated up to four months post-vaccination, reflecting mid-term durability, but long-term immune persistence remains to be determined. Additionally, the challenge experiments were conducted using a homologous strain. Future studies should examine cross-protection against drifted field strains, assess immune durability over time, and evaluate field performance under varied conditions including commercial delivery formats and genetically diverse dog populations.

Additionally, although the frameshift-based truncation of the SA1-derived NS segment likely reduced the risk of functional reversion, its genetic stability and potential for reassortment were not experimentally evaluated in this study. Furthermore, the LAIV construct does not contain engineered mutations in other segments to actively prevent reassortment with wild-type viruses. Future investigations should address these biosafety aspects through in vitro and in vivo co-infection models and rational segment design.

In conclusion, the NS1-truncated LAIV evaluated in this study offers a promising advancement in canine influenza vaccination. Through strong induction of both humoral and mucosal immunity, it provided substantial protection against clinical disease and markedly reduced viral shedding following challenge. These attributes not only improve individual animal protection but also support broader transmission control—aligning with One Health objectives aimed at reducing interspecies transmission risk and enhancing pandemic preparedness at the animal-human interface.

## Supplementary Information

**Additional file 1. Epitope hotspot definition and residue-level comparison of HA sequences between LAIV and consensus CIV**. Amino acid positions corresponding to known or predicted epitope hotspots in the HA protein were defined based on structural and antigenic mapping. For each hotspot, the central residue and its flanking ±5 residues were compared between the live attenuated influenza vaccine (LAIV) strain (consisting of 7 segments from A/canine/Korea/01/2007 (H3N2) and NS segment from A/equine/Kyonggi/SA1/2011 (H3N8)) and the consensus sequence of circulating H3N2 canine influenza virus (CIV) strains (2020–2023, NCBI registered). Substituted residues are shown in the format “CIV → consensus CIV,” with residues located at the central hotspot positions highlighted in bold. This table serves as the primary reference for subsequent structural and epitope-based evaluations in Additional file 2 and Figures 1B and 1C.

**Additional file 2. Structural and antigenic prediction of HA epitope hotspot mutations using BepiPred 2.0.** Five amino acid positions with substitutions within epitope hotspots of the HA protein—identified from the live attenuated influenza virus (LAIV) strain (consisting of 7 segments from A/canine/Korea/01/2007 (H3N2) and NS segment from A/equine/Kyonggi/SA1/2011 (H3N8)) versus consensus H3N2 canine influenza virus (CIV) strains comparison—were analyzed for potential antigenic impact using BepiPred 2.0. The changes in residue exposure (Exposed/Buried), relative surface accessibility (ΔRSA), and secondary structure probabilities (ΔHelix, ΔSheet, ΔCoil), as well as Δ epitope probability scores, are shown for each mutation. Residues exhibiting Exposed-to-Buried transitions and concurrent reductions in RSA and epitope probability were considered potential antibody evasion sites. Conversely, Buried-to-Exposed transitions or increases in coil content and epitope probability were interpreted as favorable for antibody recognition. This analysis supports structural interpretation presented in Figure 1C.

**Additional file 3. Epitope hotspot definition and residue-level comparison of NA sequences between LAIV and consensus CIV.** Epitope hotspot positions within the NA protein were selected based on antigenic loop regions and prior literature on neuraminidase immunodominant sites. For each hotspot, the central residue and its flanking ±5 residues were compared between the live attenuated influenza vaccine (LAIV) strain (consisting of 7 segments from A/canine/Korea/01/2007 (H3N2) and NS segment from A/equine/Kyonggi/SA1/2011 (H3N8)) and the consensus sequence of circulating H3N2 canine influenza virus (CIV) strains (2020–2023, NCBI registered). Substituted residues are indicated in the format “CIV → consensus CIV,” with central hotspot positions shown in bold. This comparative mapping serves as the basis for structural and epitope accessibility evaluations presented in Additional file 4 and Additional file 5.

**Additional file 4. Structural and antigenic prediction of NA epitope hotspot mutations using BepiPred 2.0**. Amino acid substitutions at five epitope hotspot positions in the NA protein, identified by comparing the live attenuated influenza vaccine (LAIV) strain (consisting of 7 segments from A/canine/Korea/01/2007 (H3N2) and NS segment from A/equine/Kyonggi/SA1/2011 (H3N8)) and consensus H3N2 canine influenza virus (CIV) sequences, were analyzed using BepiPred 2.0 to assess their impact on antibody recognition. Each mutation was evaluated for changes in surface exposure (Exposed/Buried), relative surface accessibility (ΔRSA), secondary structure probability shifts (ΔHelix, ΔSheet, ΔCoil), and Δ epitope probability. Mutations exhibiting a transition from Exposed to Buried configuration with concurrent decreases in RSA and epitope probability were interpreted as potential antibody evasion sites. In contrast, residues showing increased exposure, enhanced coil content, or elevated epitope probability were considered to favor recognition by vaccine-induced antibodies. These findings are visualized in Additional file 5.

**Additional file 5. Structural comparison of the NA protein in the LAIV strain and circulating H3N2 canine influenza viruses**. Structural models of the neuraminidase (NA) protein from A/canine/Korea/01/2007 (H3N2, LAIV strain) and the 2020–2023 consensus canine influenza virus (CIV) strain (derived from NCBI-registered sequences) were predicted using AlphaFold2 and visualized with PyMOL. The consensus NA (transparent) is overlaid with the LAIV NA structure (solid). Domains are color-coded as follows: signal peptide (residues 1–20, green), stalk region (residues 21–80, orange), catalytic head domain (residues 81–469, blue), and antigenically relevant loops (residues 220–230 and 329–339, red). Catalytic site residues (118, 151, 152, 227, 276, 292, 371, 403, 406) are shown in gold. Amino acid substitutions between LAIV and consensus CIV are mapped onto the structure and represented as sticks (LAIV in purple, consensus CIV in light purple), annotated in the format “LAIV→ consensus” to indicate directional change. Residues with potential impact on epitope accessibility or structure, based on BepiPred 2.0 analysis, are emphasized.

**Additional file 6. Conservation of predicted DLA-88–restricted T cell epitopes in the NP protein of LAIV and consensus CIV strains.** Putative T cell epitopes within the nucleoprotein (NP) of H3N2 canine influenza virus (CIV) were predicted based on their potential to be presented by canine MHC class I (DLA-88) alleles. Twelve peptide regions were selected based on sequence motifs, immunogenicity profiles, and known epitope patterns in influenza NP. Amino acid sequences of these regions were compared between the live attenuated influenza vaccine (LAIV) strain (consisting of 7 segments from H3N2 CIV and NS segment from A/equine/Kyonggi/SA1/2011 (H3N8)) and the consensus H3N2 CIV (2020–2023), and substitutions were noted. Peptides with full sequence conservation are marked as “Conserved,” while those with mutations are annotated with the specific substitution and position. No mutations were found within or near the predicted T cell epitopes, suggesting preserved MHC binding motifs and cross-recognition potential of vaccine-induced T cell responses.

**Additional file 7. Structural comparison of the NP protein in the LAIV strain and circulating H3N2 canine influenza viruses.** Structural models of the nucleoprotein (NP) from A/canine/Korea/01/2007 (H3N2, LAIV strain) and the 2020–2023 consensus canine influenza virus (CIV) strain (based on NCBI-registered sequences) were predicted using AlphaFold2 and visualized in PyMOL. The transparent structure represents consensus CIV, overlaid with the solid-colored LAIV NP. The complete NP protein (residues 1–498) is shown in blue. Predicted T cell epitope regions with potential DLA-88 binding are highlighted in red and span residues 31–39, 48–56, 60–68, 135–143, 158–166, 256–266, 275–283, 305–313, 330–338, 373–381, and 480–488. Amino acid substitutions between CIV01 and the consensus NP are mapped as sticks (LAIV in purple, consensus in light purple) and annotated in the format “LAIV → consensus” to indicate directional change. No mutations were located within or adjacent to the predicted T cell epitopes.

**Additional file 8. Amino acid substitutions in HA epitope regions between the LAIV strain (A/canine/Korea/01/2007 (H3N2)) and the consensus sequence of historical H3N8 CIVs (2010–2018).** Aligned HA sequences were analyzed to identify amino acid differences at antigenic sites A–E. Listed residues correspond to positions within defined B-cell epitope hotspots. Each substitution is annotated with the original residue (H3N2) and the corresponding residue in the H3N8 consensus sequence.

**Additional file 9. Structural comparison of HA proteins between the LAIV strain A/canine/Korea/01/2007 (H3N2) and consensus H3N8 CIVs (2010–2018).** Structural comparison of HA proteins modeled using AlphaFold2 and rendered with PyMOL. The consensus H3N8 canine influenza virus (CIV) HA (transparent), which registered in NCBI (2010-2018) is overlaid with the live attenuated influenza vaccine (LAIV) HA (solid). Domains are color-coded: HA1 (residues 1–328, blue), HA2 (residues 329–566, green), epitope-associated loop and helix structures—130-loop (135–138), 190-helix (188–190), and 220-loop (221–228)—are shown in red. Antigenic sites A–E are shown in gold (site A: 121–137, 140–146; B: 155–160, 188–198; C: 53–55, 275–278; D: 96–105, 171–172; E: 62–64, 78–83, 260–262). Substituted residues in epitope hotspots are represented as sticks (CIV in purple, consensus H3N8 CIV in light purple) with directional annotations.

**Additional file 10. Structural characterization of the NS1 protein in the LAIV compared with CIV**. NS1 protein structures were modeled from the consensus amino acid sequence of H3N2 canine influenza virus (CIV) (collected between 2020–2023, NCBI) and from the A/equine/Kyonggi/SA1/2011 (H3N8) (SA1)-derived truncated NS1 used in the LAIV strain, using AlphaFold2 and visualized in PyMOL. The LAIV-derived NS1 structure is shown as a solid ribbon, and the consensus H3N2 CIV NS1 is rendered transparently for comparison. Key domains are color-coded: RNA-binding domain (residues 1–73, green), interdomain linker (74–84, yellow), and truncated effector domain (85–117 or 85-230 blue). Residues associated with known functional motifs or strain-specific substitutions in the SA1-derived truncated NS1 are shown as spheres and annotated. Notably, the nine amino acid substitutions (E96G, Q109H, V111N, A112G, G113P, S114G, L115N, C116H, I117G) represent unique residues encoded by the SA1 strain specifically, not the equine consensus. These novel C-terminal residues are absent in the full-length canine H3N2 NS1 and may contribute to the structural and functional uniqueness of the LAIV’s NS1 protein.

**Additional file 11. Daily clinical signs in dogs post-vaccination**. Comprehensive record of clinical observations in beagle dogs monitored for 9 days following vaccination or inoculation with wild-type virus. Beagle dogs (*n *= 3 per group) were assigned to five groups:
- LAIV IN (high): 10^3.5^ TCID_50_ of a live attenuated influenza vaccine (LAIV), consisting of 7 segments from A/canine/Korea/01/2007 (H3N2) and NS segment from A/equine/Kyonggi/SA1/2011 (H3N8), administered intranasally.
- LAIV IN (low): same LAIV at 10^2.5^ TCID_50_/dog, intranasally.
- Inact-CIV IM: 2^7^ HAU/dog of inactivated A/canine/Korea/01/2007 (H3N2) virus formulated with 10% (v/v) aluminum hydroxide gel adjuvant, administered intramuscularly.
- CIV IN: 10^3.5^ TCID_50_/dog of wild-type A/canine/Korea/01/2007 (H3N2), administered intranasally.
- NC (negative control): PBS only, intranasally.
Individual clinical parameters (nasal and ocular discharge (N), coughing (C), Fever (F), and lethargy (L)) were scored daily according to severity (0 = absent, 1 = mild or moderate, 2 = severe or present (Fever)) for each dog in all experimental groups: LAIV IN (high), LAIV IN (low), Inact-CIV IM, CIV IN, and NC. Rectal temperatures were recorded daily and fever was defined as temperature ≥40.5 °C. The table presents daily individual scores for each parameter (symptom (score)), as well as daily mean scores per group. This detailed clinical monitoring demonstrates that NS1-truncated LAIV administered intranasally at either dose induced no detectable clinical signs throughout the observation period, in contrast to the mild-to-moderate signs observed in dogs inoculated with wild-type CIV. These data substantiate the excellent safety profile of the NS1-truncated LAIV platform in the target species.

**Additional file 12. Daily clinical signs in beagle dogs following CIV challenge**. Beagle dogs (*n* = 3 per group) were assigned to five groups:
- LAIV IN (high): 10^3.5^ TCID_50_ of a live attenuated influenza vaccine (LAIV), consisting of 7 segments from A/canine/Korea/01/2007 (H3N2) and NS segment from A/equine/Kyonggi/SA1/2011 (H3N8), administered intranasally.
- LAIV IN (low): same LAIV at 10^2.5^ TCID_50_/dog, intranasally.
- Inact-CIV IM: 2^7^ HAU/dog of inactivated A/canine/Korea/01/2007 (H3N2) virus formulated with 10% (v/v) aluminum hydroxide gel adjuvant, administered intramuscularly.
At 120 days post-vaccination, dogs were challenged intranasally with wild type A/canine/Korea/01/2007 (H3N2) virus at 10^6^TCID_50_/dog.
- PC (positive control): unvaccinated but challenge with the same wild-type CIV.
- NC (negative control): unvaccinated and unchallenged.
Clinical signs were assessed daily for 14 days post-challenge (dpc) across the following groups: LAIV IN (high), LAIV IN (low), inactivated CIV (IM), positive control (PC), and NC. Four clinical parameters were evaluated: Nasal and ocular discharge (N), Coughing (C), Fever (F), defined as rectal temperature ≥40.5 °C, Lethargy (L). Each symptom was scored per dog per day as follows: 0 = absent, 1 = mild or moderate, 2 = severe (or present for fever). Daily scores for each dog and the group mean values are presented. The animal marked “E” in each group represents the one selected for euthanasia on dpc 14, based on median cumulative clinical score, for subsequent histopathological evaluation (see Figure 6). This table illustrates that LAIV-vaccinated dogs, particularly at the high dose, showed no clinical signs following challenge, in contrast to unvaccinated controls, which developed marked respiratory illness.

**Additional file 13. Immunohistochemical analysis of lung tissues following CIV challenge.** Beagle dogs (*n* = 3 per group) were previously vaccinated with:
- LAIV IN (high): 10^3.5^ TCID_50_ of a live attenuated influenza vaccine (LAIV), consisting of 7 segments from A/canine/Korea/01/2007 (H3N2) and a truncated NS segment from A/equine/Kyonggi/SA1/2011 (H3N8), administered intranasally.
- LAIV IN (low): same LAIV at 10^2.5^ TCID_50_/dog, intranasally.
- Inact-CIV IM: 2^7^ HAU/dog of inactivated A/canine/Korea/01/2007 (H3N2) virus formulated with 10% (v/v) aluminum hydroxide gel adjuvant, administered intramuscularly.
At 120 days post-vaccination, dogs were challenged intranasally with wild type A/canine/Korea/01/2007 (H3N2) virus at 10^6 ^TCID_50_/dog.
- PC (positive control): unvaccinated but challenge with the same wild-type CIV.
On 14 days post-challenge (dpc), one representative dog per group—selected based on the median cumulative clinical score—was humanely euthanized. The right cranial and middle lung lobes were collected, fixed in 10% neutral buffered formalin, and stained with a monoclonal antibody against the influenza NP protein. IHC signals were evaluated semi-quantitatively using a dual-criteria scoring system: severity (grade, 0-4) and extent (stage, 0-4). Final IHC scores were calculated as grade × stage. No NP-positive cells were detected in any group, indicating complete clearance of viral antigen from the lungs by 14 dpc.

## Data Availability

The data that support the findings of this study are available from the corresponding author upon reasonable request.
