# Supplementary figures and images for: Intranasal NS1-truncated live attenuated canine influenza vaccine confers superior protection compared to inactivated vaccine in beagles
Source: Vet Res. 2025 Sep 25;56:178. doi: 10.1186/s13567-025-01624-7 (PMC12465203; doi:10.1186/s13567-025-01624-7)

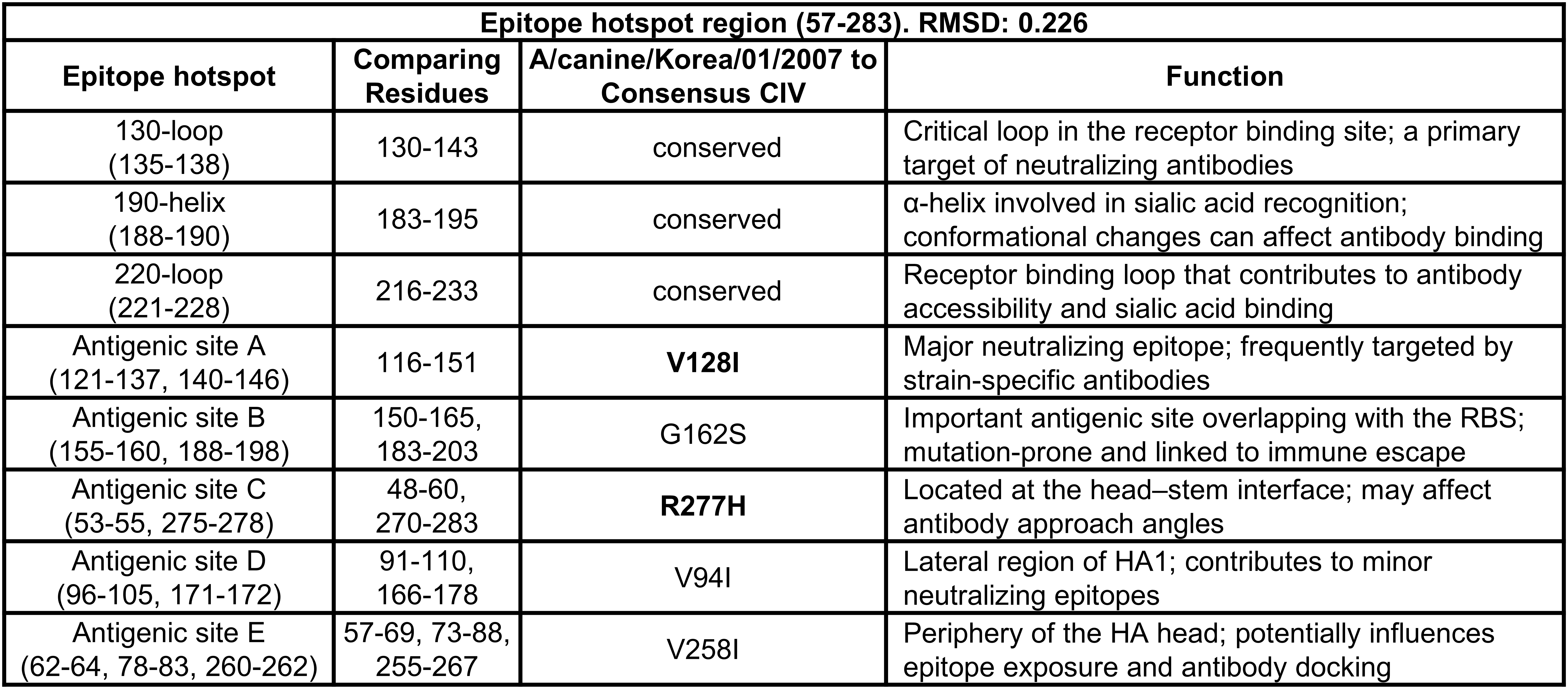

Supplement: Supplementary file 1 — Additional file 1. Epitope hotspot definition and residue-level comparison of HA sequences between LAIV and consensus CIV. Amino acid positions corresponding to known or predicted epitope hotspots in the HA protein were defined based on structural and antigenic mapping. For each hotspot, the central residue and its flanking ±5 residues were compared between the live attenuated influenza vaccine (LAIV) strain (consisting of 7 segments from A/canine/Korea/01/2007 (H3N2) and NS segment from A/equine/Kyonggi/SA1/2011 (H3N8)) and the consensus sequence of circulating H3N2 canine influenza virus (CIV) strains (2020–2023, NCBI registered). Substituted residues are shown in the format “CIV → consensus CIV,” with residues located at the central hotspot positions highlighted in bold. This table serves as the primary reference for subsequent structural and epitope-based evaluations in Additional file 2 and Figures 1B and 1C. [file 13567_2025_1624_MOESM1_ESM.tif]

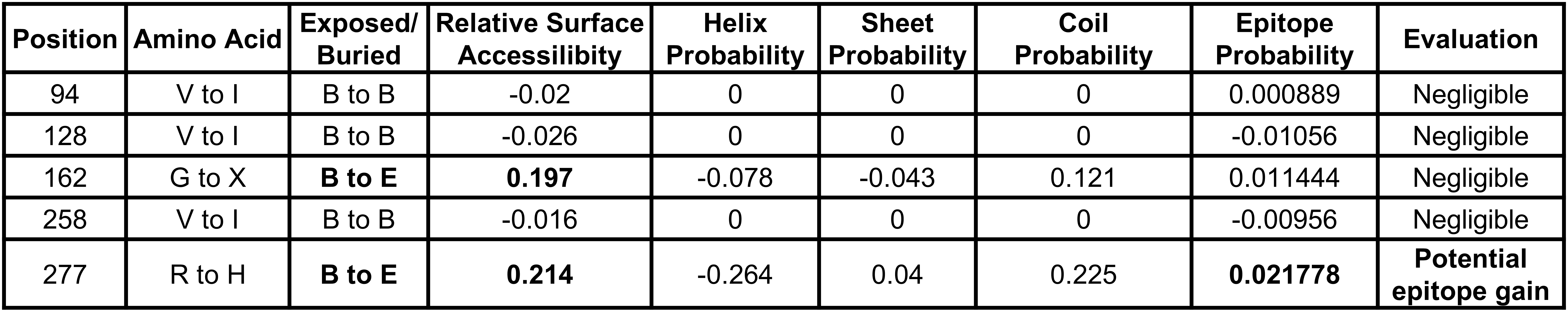

Supplement: Supplementary file 2 — Additional file 2. Structural and antigenic prediction of HA epitope hotspot mutations using BepiPred 2.0. Five amino acid positions with substitutions within epitope hotspots of the HA protein—identified from the live attenuated influenza virus (LAIV) strain (consisting of 7 segments from A/canine/Korea/01/2007 (H3N2) and NS segment from A/equine/Kyonggi/SA1/2011 (H3N8)) versus consensus H3N2 canine influenza virus (CIV) strains comparison—were analyzed for potential antigenic impact using BepiPred 2.0. The changes in residue exposure (Exposed/Buried), relative surface accessibility (ΔRSA), and secondary structure probabilities (ΔHelix, ΔSheet, ΔCoil), as well as Δ epitope probability scores, are shown for each mutation. Residues exhibiting Exposed-to-Buried transitions and concurrent reductions in RSA and epitope probability were considered potential antibody evasion sites. Conversely, Buried-to-Exposed transitions or increases in coil content and epitope probability were interpreted as favorable for antibody recognition. This analysis supports structural interpretation presented in Figure 1C. [file 13567_2025_1624_MOESM2_ESM.tif]

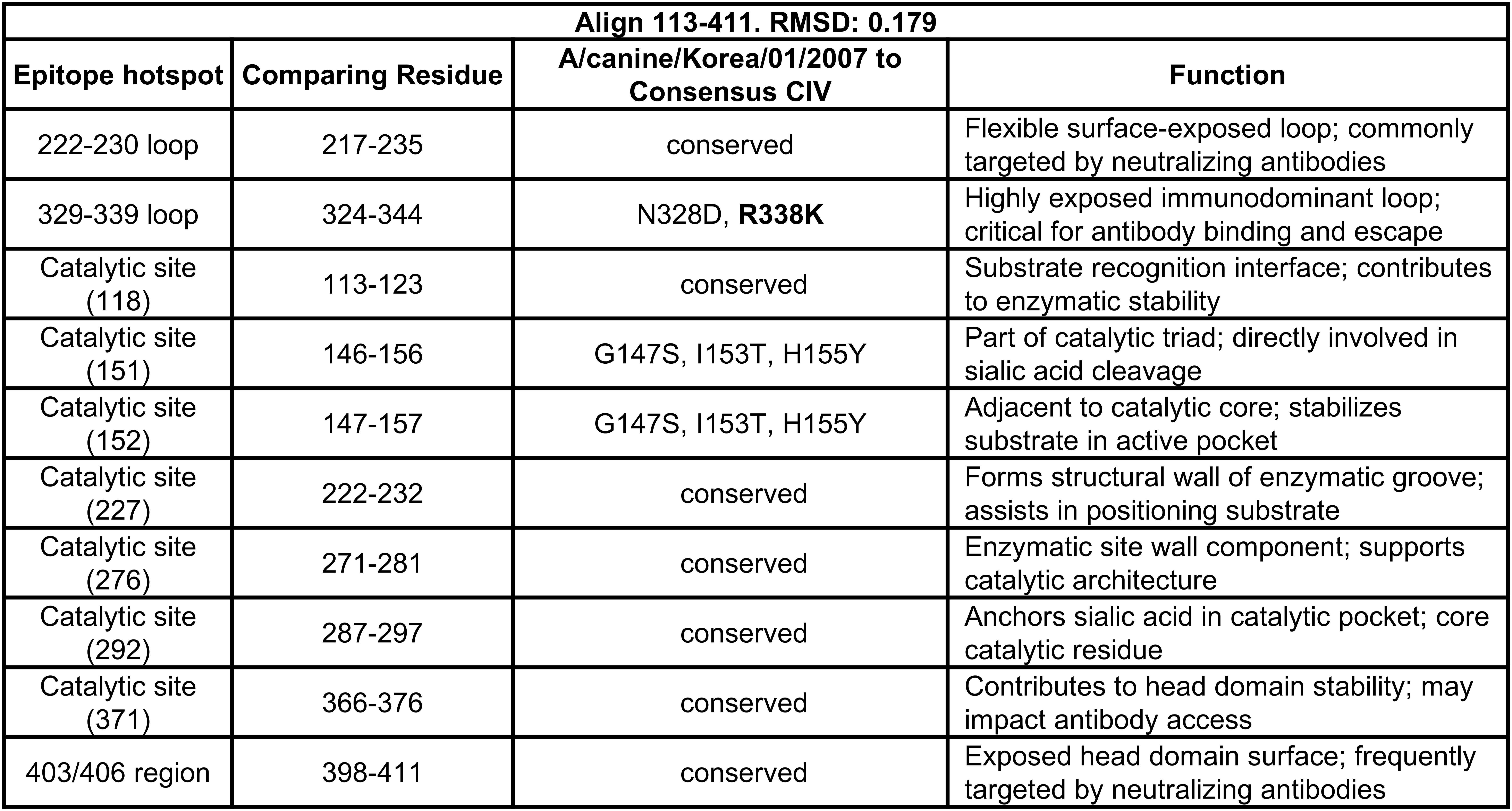

Supplement: Supplementary file 3 — Additional file 3. Epitope hotspot definition and residue-level comparison of NA sequences between LAIV and consensus CIV. Epitope hotspot positions within the NA protein were selected based on antigenic loop regions and prior literature on neuraminidase immunodominant sites. For each hotspot, the central residue and its flanking ±5 residues were compared between the live attenuated influenza vaccine (LAIV) strain (consisting of 7 segments from A/canine/Korea/01/2007 (H3N2) and NS segment from A/equine/Kyonggi/SA1/2011 (H3N8)) and the consensus sequence of circulating H3N2 canine influenza virus (CIV) strains (2020–2023, NCBI registered). Substituted residues are indicated in the format “CIV → consensus CIV,” with central hotspot positions shown in bold. This comparative mapping serves as the basis for structural and epitope accessibility evaluations presented in Additional file 4 and Additional file 5. [file 13567_2025_1624_MOESM3_ESM.tif]

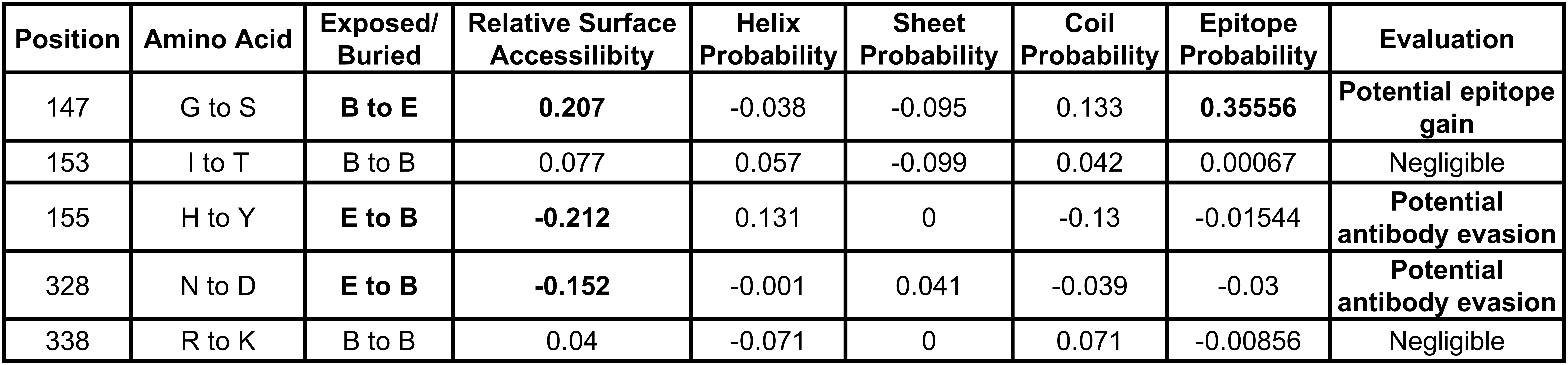

Supplement: Supplementary file 4 — Additional file 4. Structural and antigenic prediction of NA epitope hotspot mutations using BepiPred 2.0. Amino acid substitutions at five epitope hotspot positions in the NA protein, identified by comparing the live attenuated influenza vaccine (LAIV) strain (consisting of 7 segments from A/canine/Korea/01/2007 (H3N2) and NS segment from A/equine/Kyonggi/SA1/2011 (H3N8)) and consensus H3N2 canine influenza virus (CIV) sequences, were analyzed using BepiPred 2.0 to assess their impact on antibody recognition. Each mutation was evaluated for changes in surface exposure (Exposed/Buried), relative surface accessibility (ΔRSA), secondary structure probability shifts (ΔHelix, ΔSheet, ΔCoil), and Δ epitope probability. Mutations exhibiting a transition from Exposed to Buried configuration with concurrent decreases in RSA and epitope probability were interpreted as potential antibody evasion sites. In contrast, residues showing increased exposure, enhanced coil content, or elevated epitope probability were considered to favor recognition by vaccine-induced antibodies. These findings are visualized in Additional file 5. [file 13567_2025_1624_MOESM4_ESM.tif]

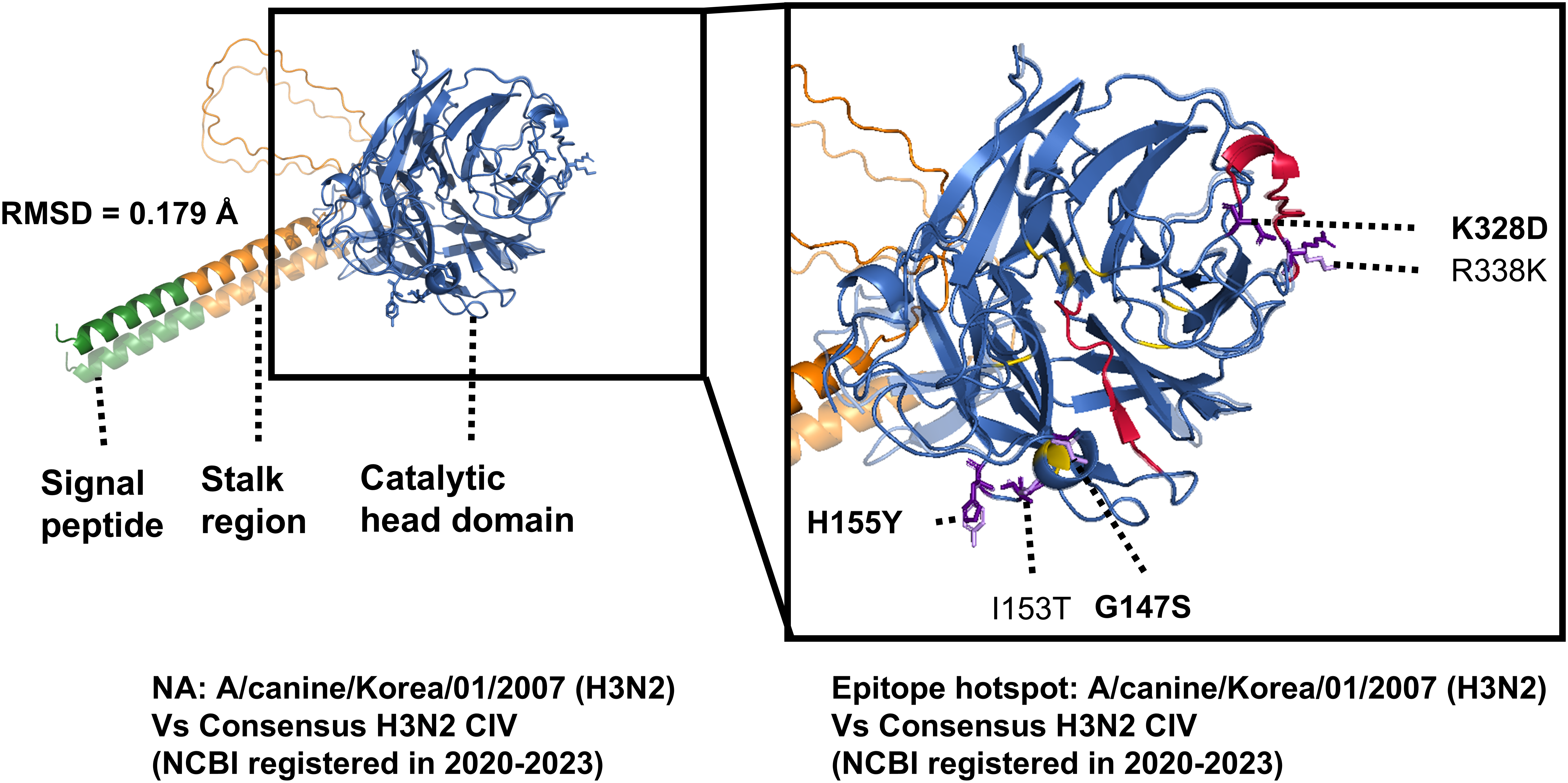

Supplement: Supplementary file 5 — Additional file 5. Structural comparison of the NA protein in the LAIV strain and circulating H3N2 canine influenza viruses. Structural models of the neuraminidase (NA) protein from A/canine/Korea/01/2007 (H3N2, LAIV strain) and the 2020–2023 consensus canine influenza virus (CIV) strain (derived from NCBI-registered sequences) were predicted using AlphaFold2 and visualized with PyMOL. The consensus NA (transparent) is overlaid with the LAIV NA structure (solid). Domains are color-coded as follows: signal peptide (residues 1–20, green), stalk region (residues 21–80, orange), catalytic head domain (residues 81–469, blue), and antigenically relevant loops (residues 220–230 and 329–339, red). Catalytic site residues (118, 151, 152, 227, 276, 292, 371, 403, 406) are shown in gold. Amino acid substitutions between LAIV and consensus CIV are mapped onto the structure and represented as sticks (LAIV in purple, consensus CIV in light purple), annotated in the format “LAIV→ consensus” to indicate directional change. Residues with potential impact on epitope accessibility or structure, based on BepiPred 2.0 analysis, are emphasized. [file 13567_2025_1624_MOESM5_ESM.tif]

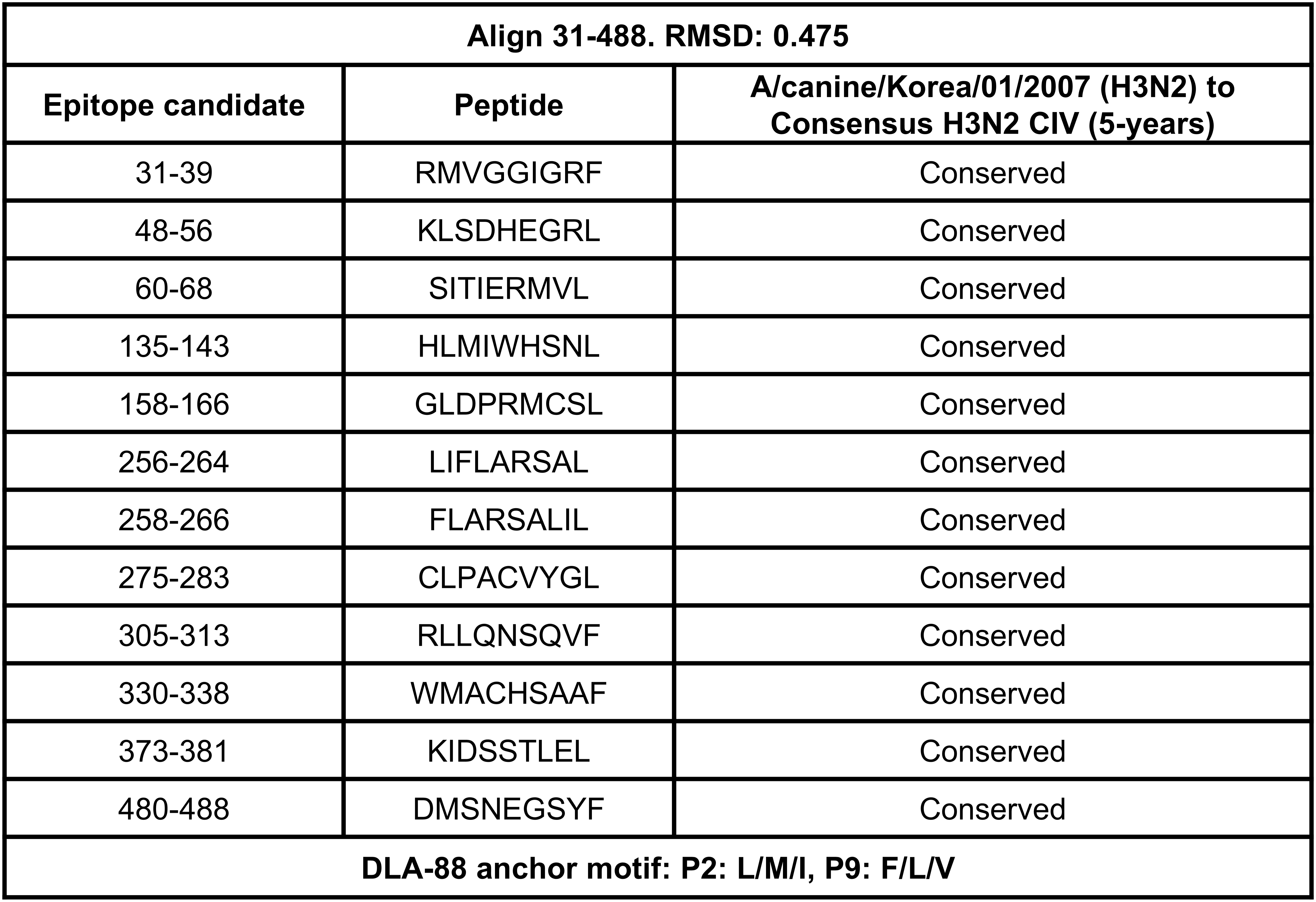

Supplement: Supplementary file 6 — Additional file 6. Conservation of predicted DLA-88–restricted T cell epitopes in the NP protein of LAIV and consensus CIV strains. Putative T cell epitopes within the nucleoprotein (NP) of H3N2 canine influenza virus (CIV) were predicted based on their potential to be presented by canine MHC class I (DLA-88) alleles. Twelve peptide regions were selected based on sequence motifs, immunogenicity profiles, and known epitope patterns in influenza NP. Amino acid sequences of these regions were compared between the live attenuated influenza vaccine (LAIV) strain (consisting of 7 segments from H3N2 CIV and NS segment from A/equine/Kyonggi/SA1/2011 (H3N8)) and the consensus H3N2 CIV (2020–2023), and substitutions were noted. Peptides with full sequence conservation are marked as “Conserved,” while those with mutations are annotated with the specific substitution and position. No mutations were found within or near the predicted T cell epitopes, suggesting preserved MHC binding motifs and cross-recognition potential of vaccine-induced T cell responses. [file 13567_2025_1624_MOESM6_ESM.tif]

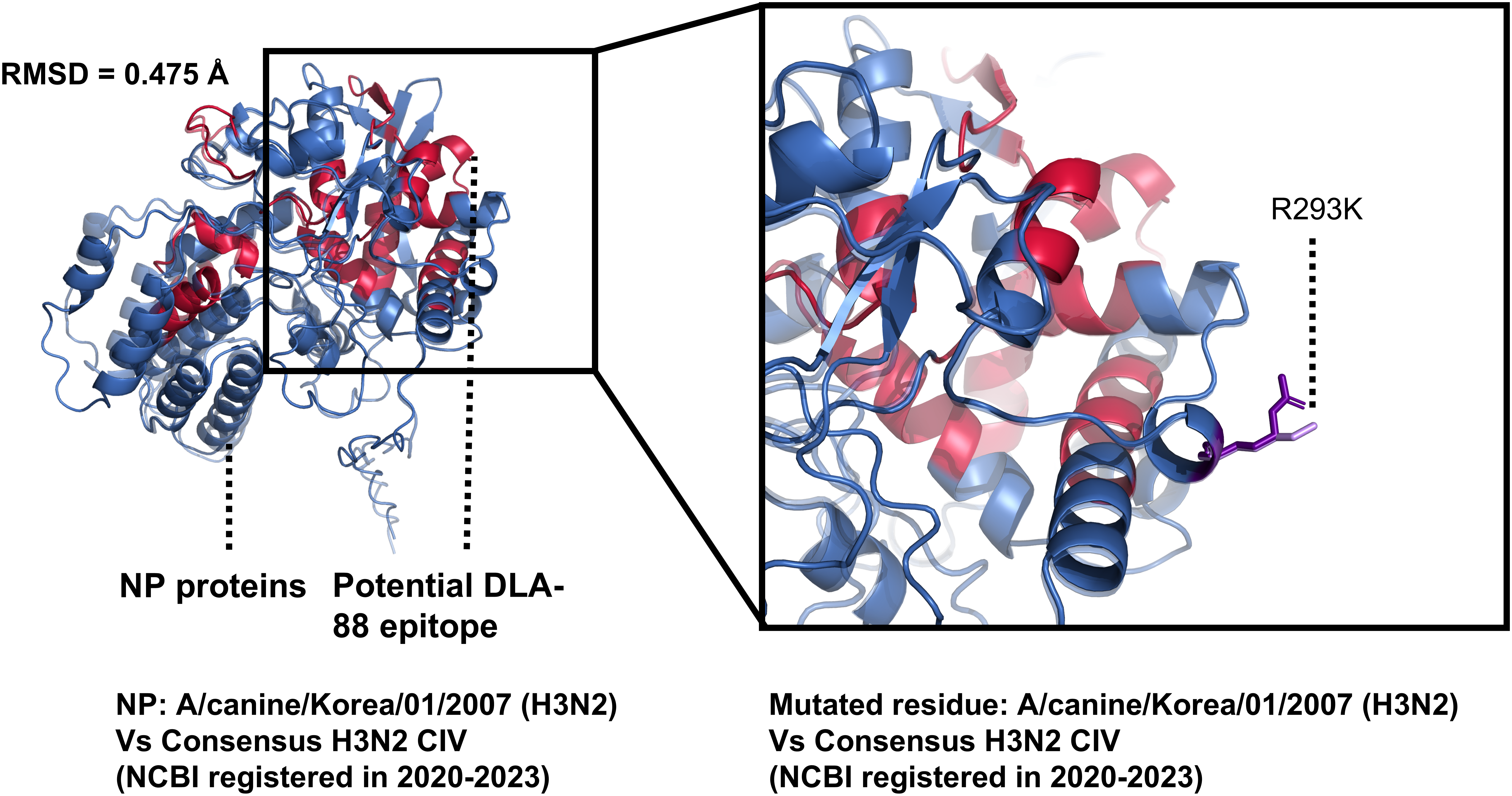

Supplement: Supplementary file 7 — Additional file 7. Structural comparison of the NP protein in the LAIV strain and circulating H3N2 canine influenza viruses. Structural models of the nucleoprotein (NP) from A/canine/Korea/01/2007 (H3N2, LAIV strain) and the 2020–2023 consensus canine influenza virus (CIV) strain (based on NCBI-registered sequences) were predicted using AlphaFold2 and visualized in PyMOL. The transparent structure represents consensus CIV, overlaid with the solid-colored LAIV NP. The complete NP protein (residues 1–498) is shown in blue. Predicted T cell epitope regions with potential DLA-88 binding are highlighted in red and span residues 31–39, 48–56, 60–68, 135–143, 158–166, 256–266, 275–283, 305–313, 330–338, 373–381, and 480–488. Amino acid substitutions between CIV01 and the consensus NP are mapped as sticks (LAIV in purple, consensus in light purple) and annotated in the format “LAIV → consensus” to indicate directional change. No mutations were located within or adjacent to the predicted T cell epitopes. [file 13567_2025_1624_MOESM7_ESM.tif]

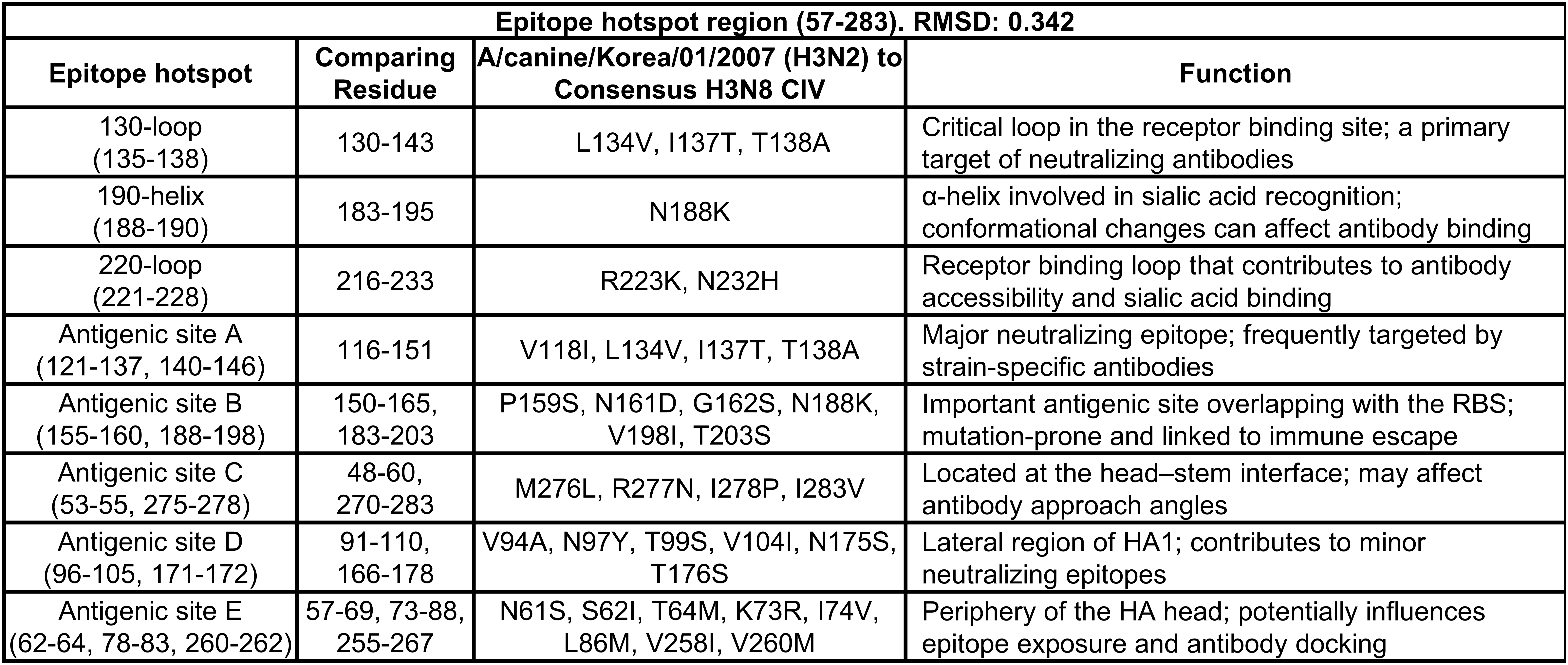

Supplement: Supplementary file 8 — Additional file 8. Amino acid substitutions in HA epitope regions between the LAIV strain (A/canine/Korea/01/2007 (H3N2)) and the consensus sequence of historical H3N8 CIVs (2010–2018). Aligned HA sequences were analyzed to identify amino acid differences at antigenic sites A–E. Listed residues correspond to positions within defined B-cell epitope hotspots. Each substitution is annotated with the original residue (H3N2) and the corresponding residue in the H3N8 consensus sequence. [file 13567_2025_1624_MOESM8_ESM.tif]

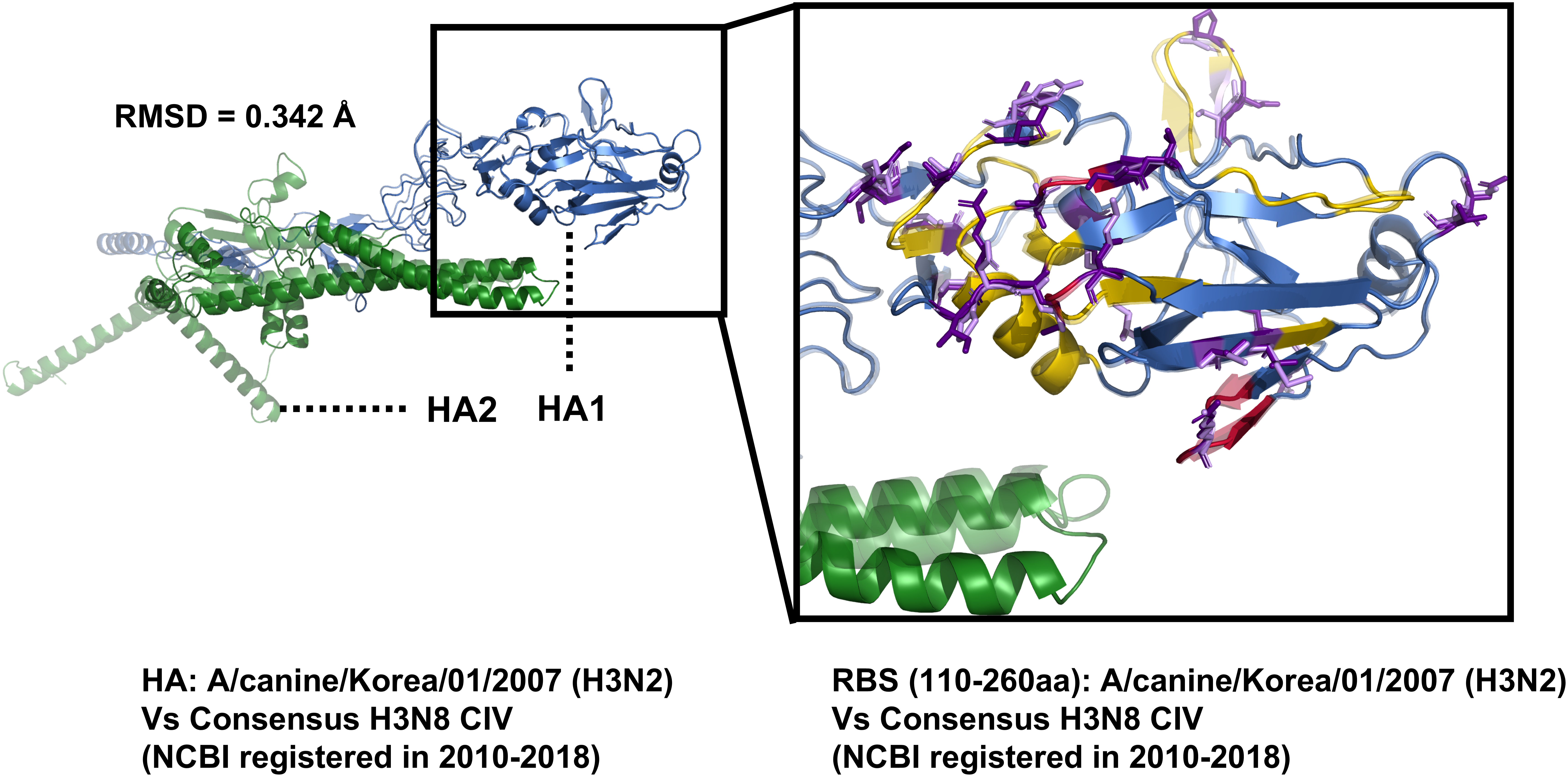

Supplement: Supplementary file 9 — Additional file 9. Structural comparison of HA proteins between the LAIV strain A/canine/Korea/01/2007 (H3N2) and consensus H3N8 CIVs (2010–2018). Structural comparison of HA proteins modeled using AlphaFold2 and rendered with PyMOL. The consensus H3N8 canine influenza virus (CIV) HA (transparent), which registered in NCBI (2010-2018) is overlaid with the live attenuated influenza vaccine (LAIV) HA (solid). Domains are color-coded: HA1 (residues 1–328, blue), HA2 (residues 329–566, green), epitope-associated loop and helix structures—130-loop (135–138), 190-helix (188–190), and 220-loop (221–228)—are shown in red. Antigenic sites A–E are shown in gold (site A: 121–137, 140–146; B: 155–160, 188–198; C: 53–55, 275–278; D: 96–105, 171–172; E: 62–64, 78–83, 260–262). Substituted residues in epitope hotspots are represented as sticks (CIV in purple, consensus H3N8 CIV in light purple) with directional annotations. [file 13567_2025_1624_MOESM9_ESM.tif]

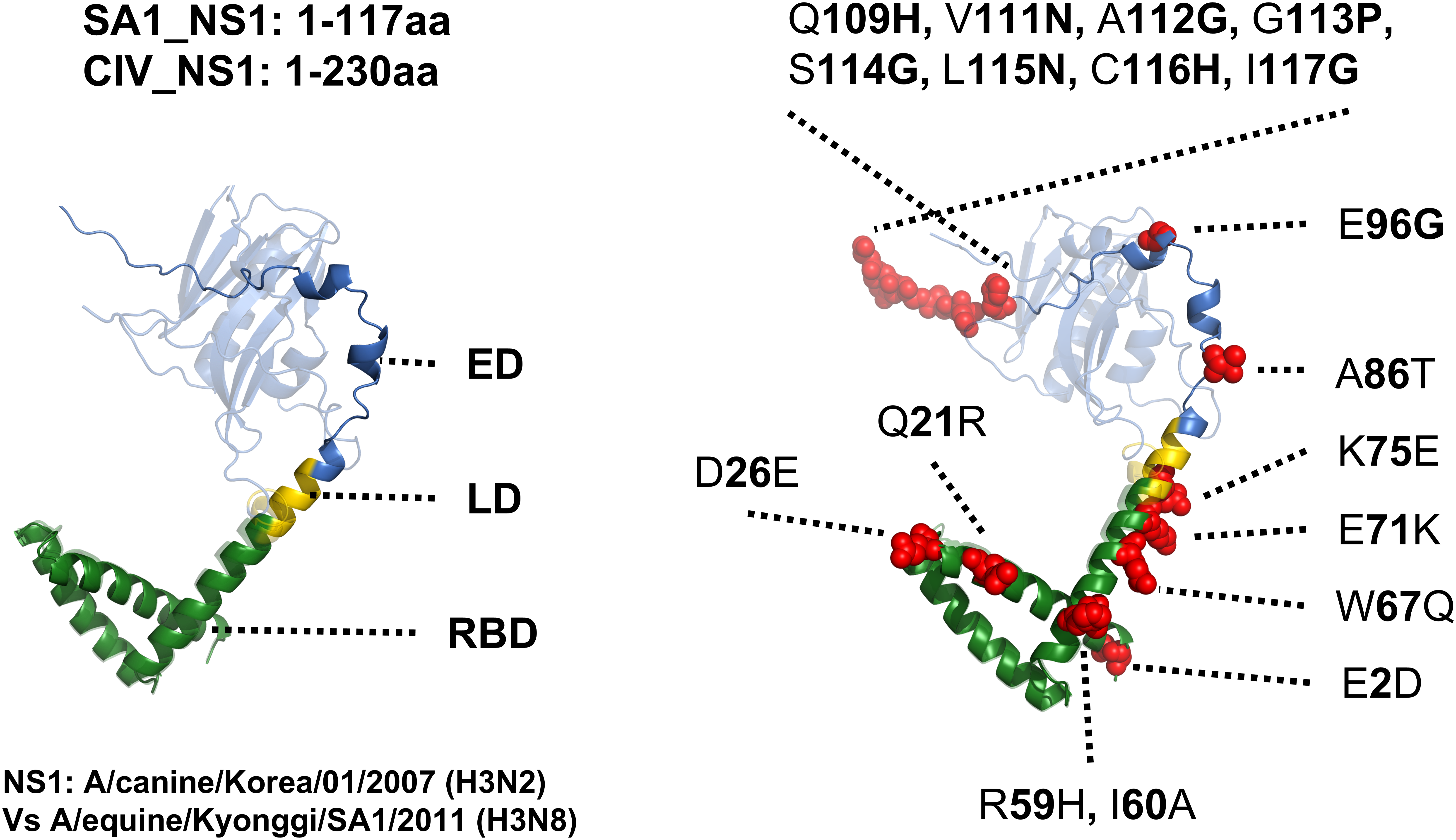

Supplement: Supplementary file 10 — Additional file 10. Structural characterization of the NS1 protein in the LAIV compared with CIV. NS1 protein structures were modeled from the consensus amino acid sequence of H3N2 canine influenza virus (CIV) (collected between 2020–2023, NCBI) and from the A/equine/Kyonggi/SA1/2011 (H3N8) (SA1)-derived truncated NS1 used in the LAIV strain, using AlphaFold2 and visualized in PyMOL. The LAIV-derived NS1 structure is shown as a solid ribbon, and the consensus H3N2 CIV NS1 is rendered transparently for comparison. Key domains are color-coded: RNA-binding domain (residues 1–73, green), interdomain linker (74–84, yellow), and truncated effector domain (85–117 or 85-230 blue). Residues associated with known functional motifs or strain-specific substitutions in the SA1-derived truncated NS1 are shown as spheres and annotated. Notably, the nine amino acid substitutions (E96G, Q109H, V111N, A112G, G113P, S114G, L115N, C116H, I117G) represent unique residues encoded by the SA1 strain specifically, not the equine consensus. These novel C-terminal residues are absent in the full-length canine H3N2 NS1 and may contribute to the structural and functional uniqueness of the LAIV’s NS1 protein. [file 13567_2025_1624_MOESM10_ESM.tif]

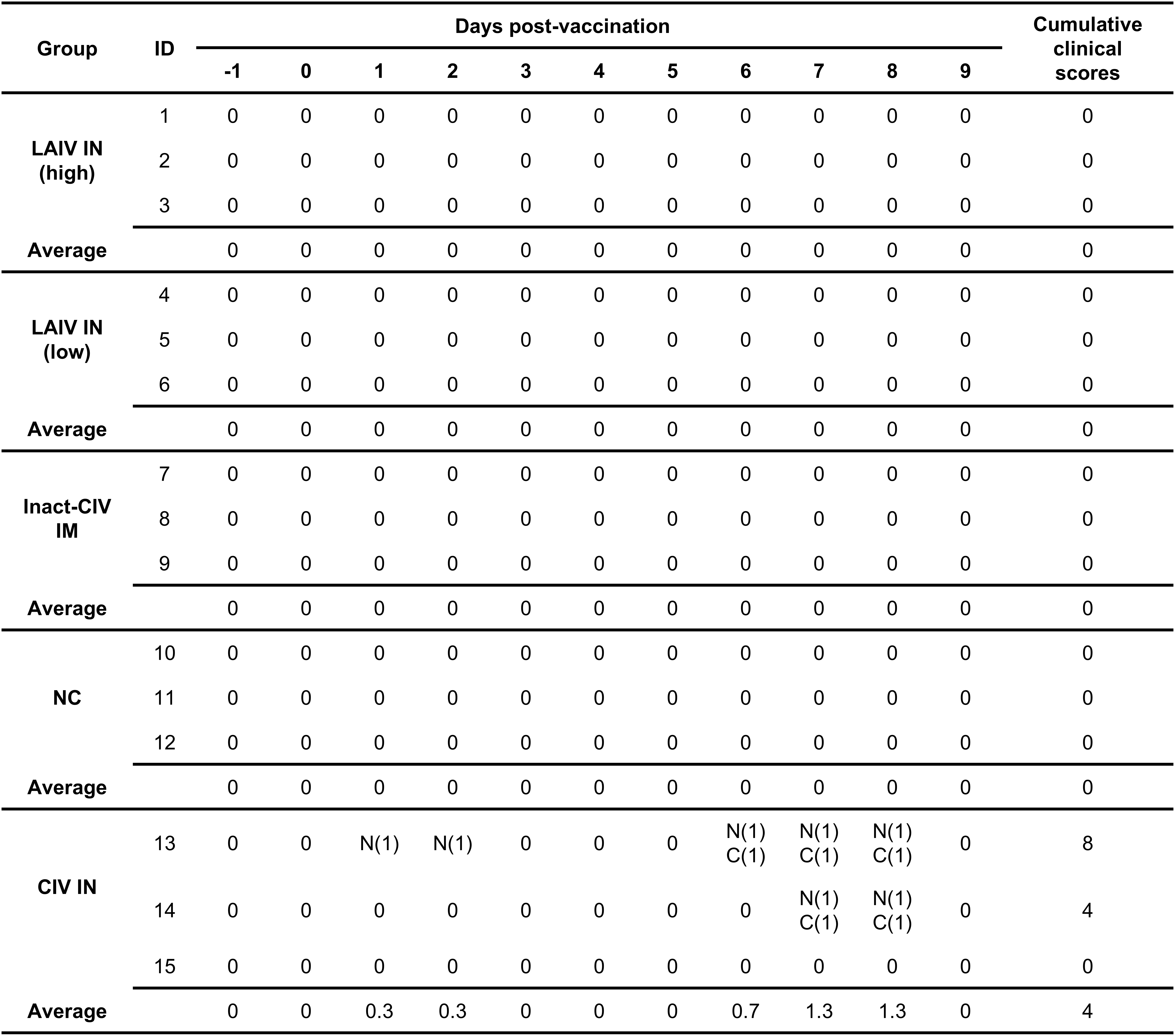

Supplement: Supplementary file 11 — Additional file 11. Daily clinical signs in dogs post-vaccination. Comprehensive record of clinical observations in beagle dogs monitored for 9 days following vaccination or inoculation with wild-type virus. Beagle dogs (n = 3 per group) were assigned to five groups: - LAIV IN (high): 103.5 TCID50 of a live attenuated influenza vaccine (LAIV), consisting of 7 segments from A/canine/Korea/01/2007 (H3N2) and NS segment from A/equine/Kyonggi/SA1/2011 (H3N8), administered intranasally. - LAIV IN (low): same LAIV at 102.5 TCID50/dog, intranasally. - Inact-CIV IM: 27 HAU/dog of inactivated A/canine/Korea/01/2007 (H3N2) virus formulated with 10% (v/v) aluminum hydroxide gel adjuvant, administered intramuscularly. - CIV IN: 103.5 TCID50/dog of wild-type A/canine/Korea/01/2007 (H3N2), administered intranasally. - NC (negative control): PBS only, intranasally. Individual clinical parameters (nasal and ocular discharge (N), coughing (C), Fever (F), and lethargy (L)) were scored daily according to severity (0 = absent, 1 = mild or moderate, 2 = severe or present (Fever)) for each dog in all experimental groups: LAIV IN (high), LAIV IN (low), Inact-CIV IM, CIV IN, and NC. Rectal temperatures were recorded daily and fever was defined as temperature ≥40.5 °C. The table presents daily individual scores for each parameter (symptom (score)), as well as daily mean scores per group. This detailed clinical monitoring demonstrates that NS1-truncated LAIV administered intranasally at either dose induced no detectable clinical signs throughout the observation period, in contrast to the mild-to-moderate signs observed in dogs inoculated with wild-type CIV. These data substantiate the excellent safety profile of the NS1-truncated LAIV platform in the target species. [file 13567_2025_1624_MOESM11_ESM.tif]

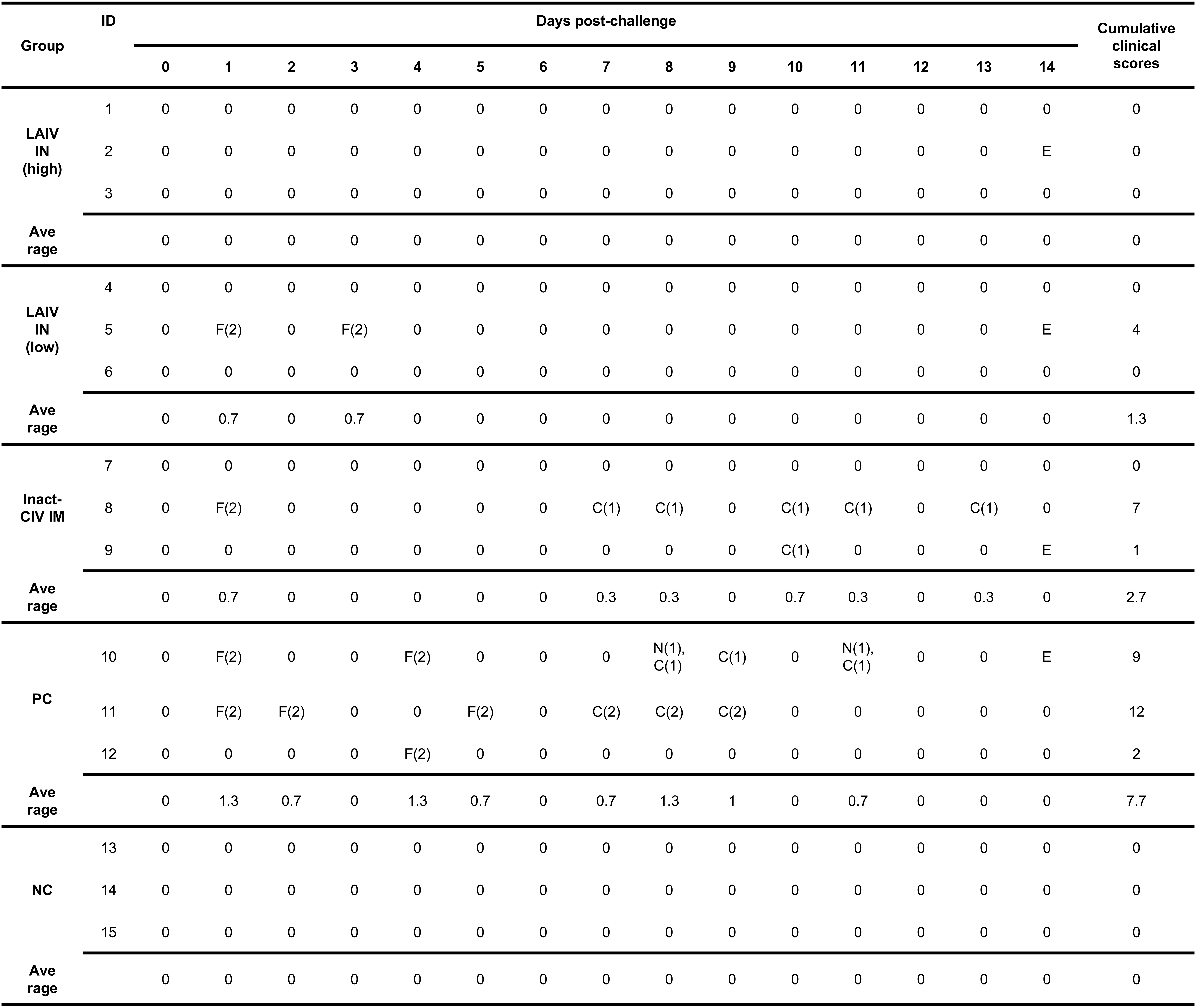

Supplement: Supplementary file 12 — Additional file 12. Daily clinical signs in beagle dogs following CIV challenge. Beagle dogs (n = 3 per group) were assigned to five groups: - LAIV IN (high): 103.5 TCID50 of a live attenuated influenza vaccine (LAIV), consisting of 7 segments from A/canine/Korea/01/2007 (H3N2) and NS segment from A/equine/Kyonggi/SA1/2011 (H3N8), administered intranasally. - LAIV IN (low): same LAIV at 102.5 TCID50/dog, intranasally. - Inact-CIV IM: 27 HAU/dog of inactivated A/canine/Korea/01/2007 (H3N2) virus formulated with 10% (v/v) aluminum hydroxide gel adjuvant, administered intramuscularly. At 120 days post-vaccination, dogs were challenged intranasally with wild type A/canine/Korea/01/2007 (H3N2) virus at 106TCID50/dog. - PC (positive control): unvaccinated but challenge with the same wild-type CIV. - NC (negative control): unvaccinated and unchallenged. Clinical signs were assessed daily for 14 days post-challenge (dpc) across the following groups: LAIV IN (high), LAIV IN (low), inactivated CIV (IM), positive control (PC), and NC. Four clinical parameters were evaluated: Nasal and ocular discharge (N), Coughing (C), Fever (F), defined as rectal temperature ≥40.5 °C, Lethargy (L). Each symptom was scored per dog per day as follows: 0 = absent, 1 = mild or moderate, 2 = severe (or present for fever). Daily scores for each dog and the group mean values are presented. The animal marked “E” in each group represents the one selected for euthanasia on dpc 14, based on median cumulative clinical score, for subsequent histopathological evaluation (see Figure 6). This table illustrates that LAIV-vaccinated dogs, particularly at the high dose, showed no clinical signs following challenge, in contrast to unvaccinated controls, which developed marked respiratory illness. [file 13567_2025_1624_MOESM12_ESM.tif]

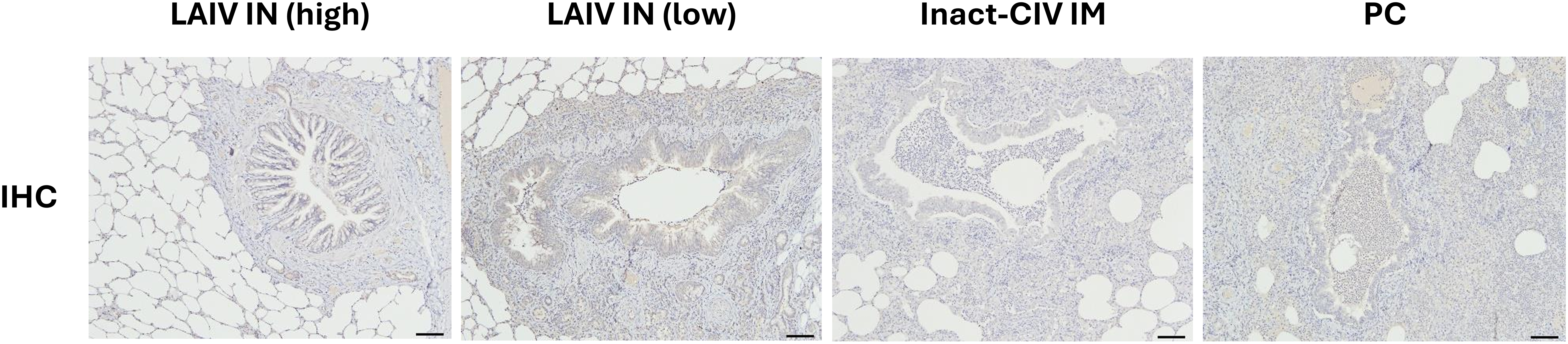

Supplement: Supplementary file 13 — Additional file 13. Immunohistochemical analysis of lung tissues following CIV challenge. Beagle dogs (n = 3 per group) were previously vaccinated with: - LAIV IN (high): 103.5 TCID50 of a live attenuated influenza vaccine (LAIV), consisting of 7 segments from A/canine/Korea/01/2007 (H3N2) and a truncated NS segment from A/equine/Kyonggi/SA1/2011 (H3N8), administered intranasally. - LAIV IN (low): same LAIV at 102.5 TCID50/dog, intranasally. - Inact-CIV IM: 27 HAU/dog of inactivated A/canine/Korea/01/2007 (H3N2) virus formulated with 10% (v/v) aluminum hydroxide gel adjuvant, administered intramuscularly. At 120 days post-vaccination, dogs were challenged intranasally with wild type A/canine/Korea/01/2007 (H3N2) virus at 106 TCID50/dog. - PC (positive control): unvaccinated but challenge with the same wild-type CIV. On 14 days post-challenge (dpc), one representative dog per group—selected based on the median cumulative clinical score—was humanely euthanized. The right cranial and middle lung lobes were collected, fixed in 10% neutral buffered formalin, and stained with a monoclonal antibody against the influenza NP protein. IHC signals were evaluated semi-quantitatively using a dual-criteria scoring system: severity (grade, 0-4) and extent (stage, 0-4). Final IHC scores were calculated as grade × stage. No NP-positive cells were detected in any group, indicating complete clearance of viral antigen from the lungs by 14 dpc. [file 13567_2025_1624_MOESM13_ESM.tif]
